# A Theoretical Hypothesis on How Immune Cells May Transmit Acquired Traits: A Macrophage–piRNA Pathway for Transgenerational Inheritance

**DOI:** 10.3390/cells15111030

**Published:** 2026-06-03

**Authors:** Douglas M. Ruden

**Affiliations:** Department of Obstetrics and Gynecology, Charles S. Mott Center for Human Growth and Development, Institute of Environmental Health Sciences, Wayne State University, Detroit, MI 48201, USA; douglasr@wayne.edu; Tel.: +1-313-577-6688

**Keywords:** piRNAs, transgenerational epigenetic inheritance, macrophages, extracellular vesicles, environmental stress, Hsp90, transposons, small non-coding RNAs, macrophage polarization, epigenetic inheritance

## Abstract

Environmental exposures can influence phenotypes across generations, yet the cellular routes by which somatic stress signals reach the germline remain poorly defined. piRNAs are attractive candidates for transgenerational epigenetic inheritance because they silence transposable elements, guide chromatin regulation, carry a stabilizing 3′ 2′-O-methyl modification, and participate in self-reinforcing amplification pathways, including ping-pong amplification in animals and RNA-dependent RNA polymerase (RdRP)-mediated secondary small-RNA amplification in systems such as *C. elegans*. This review examines evidence linking piRNAs, macrophage biology, and environmentally induced inheritance. We first summarize small-RNA inheritance in animals, plants, and ciliates, emphasizing *C. elegans* piRNA-triggered epigenetic memory and plant RNA-directed DNA methylation as parallel small-RNA-based inheritance systems. We then discuss emerging evidence that macrophage polarization states contain distinct piRNA signatures and release extracellular vesicles carrying non-coding RNAs. Finally, we revisit the *Drosophila* ectopic large bristle outgrowth (ELBO) phenotype as a possible example of macrophage-like hemocytes linking stress, tissue remodeling, and heritable morphological variation. We propose the macrophage-mediated morphological evolution (M3) model as a testable framework connecting environmental stress to transgenerational phenotypes.

## 1. Introduction

The mechanisms by which environmental experiences influence phenotypes across generations remain one of the most debated questions in modern biology. Classical evolutionary theory, as formalized in the modern evolutionary synthesis, proposes that heritable variation arises primarily through stochastic genetic mutation followed by natural selection [1,2,3,4]. However, accumulating evidence from plants, invertebrates, and mammals suggests that environmental stressors—including nutritional changes, toxicant exposures, inflammation, and psychological stress—can induce phenotypic changes that persist across generations in the absence of detectable DNA sequence alterations [5,6,7,8]. These observations have renewed interest in mechanisms of transgenerational epigenetic inheritance and raise important questions regarding how environmentally induced information is transmitted from somatic tissues to the germline [8,9,10,11].

Among the molecular systems implicated in transgenerational inheritance, small non-coding RNAs have emerged as particularly important regulators [12,13]. These include microRNAs (miRNAs) [12,14], small interfering RNAs (siRNAs) [15], tRNA-derived fragments (tRFs) [16,17,18,19], and PIWI-interacting RNAs (piRNAs) [20]. piRNAs are especially intriguing because they function in genome defense by silencing transposable elements and guiding chromatin modifications in association with PIWI-family proteins [21,22]. Unlike miRNAs, piRNAs are generally longer, possess a characteristic 2′-O-methyl modification at their 3′ end that increases their stability, and participate in self-amplifying “ping-pong” amplification pathways that can reinforce and propagate silencing signals [23,24,25]. In organisms such as *C. elegans*, piRNA-triggered pathways can initiate transgenerational gene-silencing programs that persist for multiple generations through interactions with secondary small RNAs and chromatin regulatory systems [26,27,28,29]. Related small-RNA-directed inheritance systems also occur in plants through RNA-directed DNA methylation (RDDM) pathways [30] and in ciliates through small-RNA-guided genome rearrangement and DNA elimination mechanisms [31,32,33].

Despite growing evidence that small RNAs contribute to epigenetic inheritance, the cellular pathways that connect stressed somatic tissues to germline cells remain poorly understood. Several studies suggest that sperm small-RNA populations are altered following environmental exposures such as toxicant exposure, dietary stress, and inflammation [10,34,35,36,37,38]. tRNA-derived fragments have received particular attention in this context because altered tRF profiles have been associated with paternal dietary and environmental exposures [16,39,40,41]. However, the mechanisms by which transient tRF signals might be stably propagated across multiple generations remain unclear, particularly because no amplification mechanism analogous to the piRNA ping-pong cycle has yet been identified for tRFs [42]. Similarly, recent studies in *C. elegans* demonstrate that somatic tissues can communicate directly with the germline through epigenetic mechanisms, where intestine-derived histone H3.3 is transported into germ cells and modified to encode transgenerational memory of metabolic stress, thereby linking somatic environmental sensing to heritable phenotypic outcomes [29]. In contrast to tRFs and recently described soma-to-germline histone H3.3 signaling pathways [29], which transmit environmental information without clear mechanisms for amplification, piRNA pathways possess intrinsic self-amplifying and chromatin-targeting properties that make them particularly well suited for stable transgenerational inheritance mechanisms [43].

Recent studies have also begun to identify unexpected links between small-RNA biology and immune-cell function. Macrophages and macrophage-like phagocytic cells are highly plastic immune cells involved in inflammation, tissue remodeling, wound healing, and developmental tissue sculpting [44,45]. These cells can adopt distinct activation states, broadly classified as pro-inflammatory M1 and tissue-remodeling M2 phenotypes [46]. Importantly, recent small-RNA sequencing studies demonstrate that macrophage polarization states contain distinct piRNA expression profiles [47]. Specific piRNAs have been linked to regulation of inflammatory signaling pathways, including TNF-α and HIF-1α signaling axes involved in macrophage activation and tissue remodeling [47]. In addition, macrophages release extracellular vesicles containing diverse classes of small non-coding RNAs, including piRNAs, suggesting that macrophages may participate in systemic RNA-based communication pathways [47,48,49].

The possibility that immune cells participate in transgenerational inheritance has received relatively little attention, even though macrophages possess several characteristics that make them plausible mediators of environmentally induced epigenetic signaling. Macrophages migrate extensively through tissues, phagocytose dying cells, integrate inflammatory and developmental signals, and release extracellular vesicles capable of transporting RNA cargo to distant tissues [50,51,52]. These properties raise the possibility that macrophages may act as mobile carriers of stress-associated small RNAs capable of transmitting environmentally induced signals to germline cells.

This review examines emerging evidence linking macrophages, piRNAs, environmental stress responses, and transgenerational epigenetic inheritance. We first summarize current understanding of piRNA biology and small-RNA-mediated inheritance systems across animals, plants, and other eukaryotes. We then review evidence linking environmental stress, Hsp90 signaling, and transposon activation to epigenetic instability and phenotypic variation. Next, we discuss recent findings demonstrating distinct piRNA signatures in macrophage polarization states and the role of macrophage-derived extracellular vesicles in RNA-mediated communication. We also revisit earlier studies initiated in my laboratory over 30 years ago on the ectopic large bristle outgrowth (ELBO) phenotype in *Drosophila melanogaster* as a potential example of macrophage-associated morphological inheritance mediated by stress-responsive signaling pathways [53,54,55,56]. Finally, we propose the macrophage-mediated morphological evolution (M3) model as a conceptual framework linking environmental stress, macrophage biology, piRNA signaling, and transgenerational phenotypic inheritance.

The central hypothesis explored in this review is that macrophages and related phagocytic immune cells may function as intermediaries between stressed somatic tissues and the germline by acquiring, amplifying, and systemically distributing stress-responsive piRNAs and other small RNAs. If correct, this model would suggest that immune systems contribute not only to pathogen defense and tissue homeostasis but also to the generation and transmission of environmentally responsive phenotypic variation across generations.

## 2. Methods

**Figure 1:**
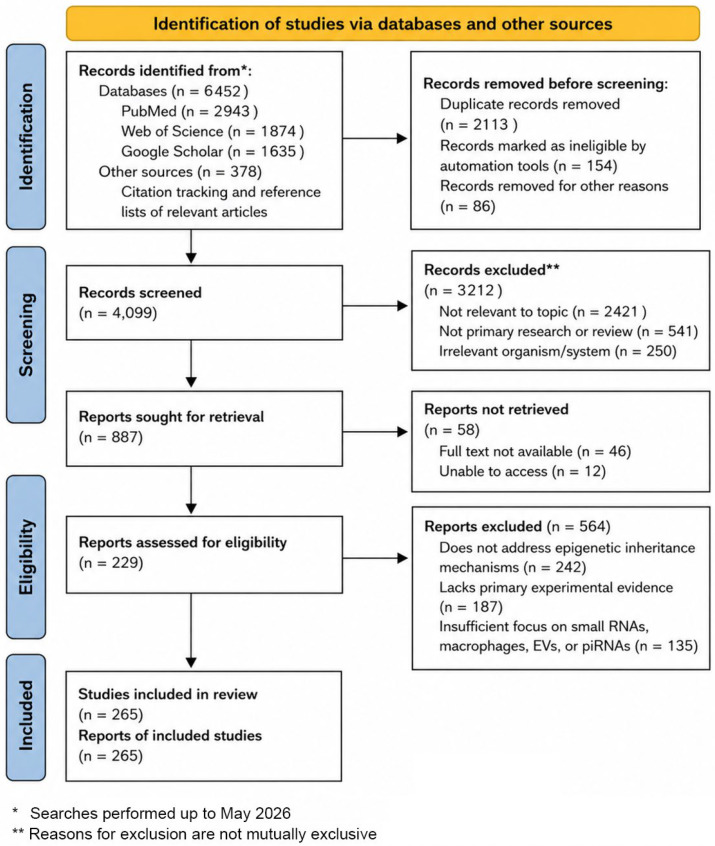
PRISMA diagram for this theoretical hypothesis paper. PRISMA stands for Preferred Reporting Items for Systematic Reviews and Meta-Analyses. It refers to a standardized framework used to transparently report how studies were identified, screened, selected, and included in a systematic review.

This review was conducted as a mechanistic and conceptual synthesis of the literature examining potential links between piRNAs, macrophage biology, extracellular vesicle signaling, environmental stress responses, and transgenerational epigenetic inheritance. Because the field spans developmental biology, immunology, epigenetics, evolutionary biology, and environmental health sciences, the reviewed studies include heterogeneous experimental systems ranging from *Drosophila* and *C. elegans* to mammalian and human models. Accordingly, the review was designed as a structured narrative review, conducting a systematic literature search of components rather than a quantitative meta-analysis or clinical evidence assessment. Literature searches were performed using PubMed, Google Scholar, and Web of Science databases for articles published through 1 May 2026. Search combinations included the following terms: “piRNA”, “PIWI”, “macrophage polarization”, “M1 macrophage”, “M2 macrophage”, “extracellular vesicles”, “exosomes”, “transgenerational epigenetic inheritance”, “small non-coding RNA”, “tRNA fragments”, “Hsp90”, “transposons”, “environmental stress”, “hemocytes”, “Drosophila”, “*C. elegans*”, “RNA-directed DNA methylation”, and “germline inheritance”. Additional studies were identified through citation tracking of primary research articles and review papers. Preference was given to peer-reviewed experimental studies providing mechanistic insights into small-RNA biology, chromatin regulation, macrophage function, extracellular vesicle signaling, developmental remodeling, stress biology, and transgenerational inheritance systems. Studies involving macrophage-derived extracellular vesicles, PIWI/piRNA pathway regulation, sperm epigenetics, transposon silencing, and environmentally induced phenotypic inheritance were prioritized for detailed analysis. Because the reviewed studies used diverse experimental designs and biological systems, formal risk-of-bias scoring frameworks commonly used in clinical systematic reviews were not uniformly applicable. Instead, emphasis was placed on reproducibility across independent experimental systems, mechanistic evidence, and consistency with established biological pathways. Articles not directly related to epigenetic inheritance mechanisms or lacking primary experimental evidence were generally excluded unless they provided important historical, conceptual, or evolutionary context relevant to the proposed macrophage-mediated morphological (M3) model (Figure 1). This theoretical hypothesis paper is registered with the Open Science Framework (OSF): Registration DOI 10.17605/OSF.IO/XF6BC.

## 3. Results

This section is divided into thematic subheadings summarizing current evidence linking piRNAs, macrophage biology, environmental stress responses, and transgenerational epigenetic inheritance. The reviewed findings support a conceptual framework in which macrophage-associated small RNAs may contribute to the transmission of environmentally induced phenotypes across generations.

### 3.1. piRNAs as Mediators of Epigenetic Inheritance

#### 3.1.1. Canonical Functions of piRNAs in Genome Defense

**Figure 2:**
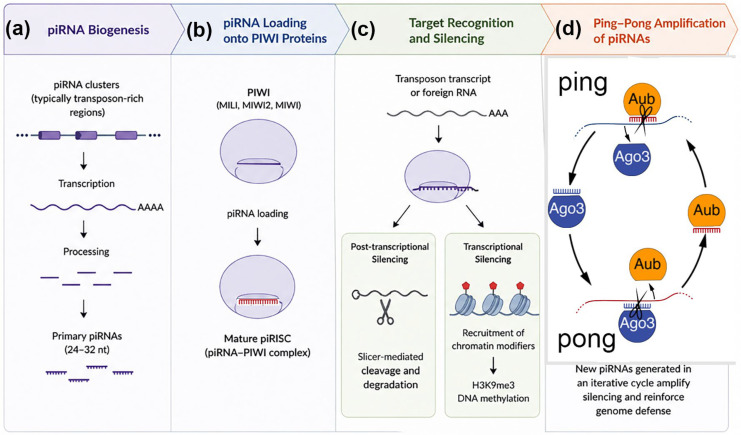
Canonical functions of piRNAs in genome defense. This graphic abstract summarizes the major roles of PIWI-interacting RNAs (piRNAs) in genome defense and maintenance of genomic stability. (**a**) piRNA biogenesis. piRNAs are generated from transposon-rich genomic regions known as piRNA clusters. These precursor transcripts are processed into mature primary piRNAs, typically 24–32 nucleotides in length. (**b**) Loading onto PIWI proteins. Mature piRNAs associate with PIWI-family proteins to form piRNA-induced silencing complexes (piRISCs). These complexes function as sequence-specific guides that recognize complementary transposon-derived or foreign RNA transcripts. MIWI (Mouse PIWI) is one of the specific PIWI-family proteins found in mice, essential for male fertility. MILI stands for “Mouse PILI” (meaning “like PIWI”) and is the homolog of the human PIWI-like 2 gene (PIWIL2). (**c**) Target recognition and silencing. piRNA–PIWI complexes mediate both post-transcriptional and transcriptional silencing pathways. In the cytoplasm, target RNAs are cleaved and degraded through slicer-dependent mechanisms. In the nucleus, piRNA pathways recruit chromatin-modifying complexes that establish repressive epigenetic marks, including histone H3 lysine 9 trimethylation (H3K9me3) and DNA methylation, resulting in heterochromatin formation and stable transcriptional repression of transposable elements. (**d**) Ping-pong amplification cycle. Cytoplasmic PIWI proteins participate in a self-reinforcing “ping-pong” amplification pathway in which cleavage of transposon transcripts generates secondary piRNAs that further amplify silencing responses. This amplification system strengthens genome defense and contributes to maintenance of heritable epigenetic states. Aubergine (Aub) and Argonaut 3 (Ago3) are cytoplasmic PIWI family members. Together, these pathways protect genome integrity by repressing transposable elements, preventing insertional mutagenesis and chromosomal instability, and preserving germline genome stability across generations. The evolutionary conservation of piRNA pathways across animals suggests that these mechanisms represent ancient genome-defense systems that may also contribute to transgenerational epigenetic inheritance.

PIWI-interacting RNAs (piRNAs) are a specialized class of small non-coding RNAs that play essential roles in genome defense, particularly through the repression of transposable elements in germline cells (Figure 2). Unlike microRNAs (miRNAs) and small interfering RNAs (siRNAs), piRNAs are generally 24–32 nucleotides in length and associate specifically with PIWI-family proteins, a specialized subfamily of Argonaute proteins [57,58,59]. piRNAs are highly enriched in animal germlines, where they function to preserve genome integrity during gametogenesis by preventing the mobilization of transposable elements, which can otherwise induce insertional mutagenesis, chromosome instability, and epigenetic disruption [60,61].

One of the primary functions of piRNAs is the sequence-specific silencing of transposable elements at both transcriptional and post-transcriptional levels. In the nucleus, PIWI–piRNA complexes recognize transposon-derived sequences and recruit chromatin-modifying machinery that establishes repressive epigenetic marks, including histone H3 lysine 9 trimethylation (H3K9me3), leading to heterochromatin formation and transcriptional silencing of repetitive elements [62,63]. In mammals, these pathways are also linked to DNA methylation of transposable elements during germline development, further stabilizing transposon repression across cell divisions [64,65]. Through these mechanisms, piRNAs contribute not only to immediate genome defense but also to the maintenance of heritable epigenetic states that can persist across generations.

In addition to their nuclear functions, piRNAs participate in post-transcriptional silencing pathways within the cytoplasm. Cytoplasmic PIWI-family proteins such as Aubergine and Argonaute3 in Drosophila participate in the “ping-pong” amplification cycle, a self-reinforcing mechanism that amplifies piRNA populations through reciprocal cleavage of transposon-derived transcripts [66,67]. In this pathway, piRNA-guided cleavage of transposon RNAs generates additional piRNAs that can subsequently load onto complementary PIWI proteins, thereby reinforcing transposon silencing [67]. This amplification system is particularly important because it provides a plausible mechanism for the propagation and maintenance of small-RNA-mediated epigenetic information across developmental stages and potentially across generations.

Another distinctive feature of piRNAs is their 3′ terminal 2′-O-methyl modification, which increases RNA stability and resistance to exonuclease degradation [24,68]. This chemical modification distinguishes piRNAs from many other small RNAs and may contribute to their suitability for long-term epigenetic signaling and inheritance. Importantly, the 2′-O-methyl modification also allows piRNAs to be selectively enriched experimentally using sodium periodate oxidation protocols, which preferentially degrade non-methylated RNAs while preserving mature piRNAs [24]. Despite these unique properties, piRNAs remain comparatively understudied in many somatic tissues relative to miRNAs and tRNA-derived fragments.

Although piRNA pathways were initially thought to function almost exclusively in germline cells, increasing evidence indicates that PIWI proteins and piRNAs also operate in selected somatic tissues, including stem cells, cancer cells, and immune cells [25,26,27]. These observations raise the possibility that piRNA pathways may have broader biological functions beyond germline genome defense, including roles in environmental stress responses, chromatin remodeling, and intercellular communication. Such findings are particularly relevant to emerging models of transgenerational epigenetic inheritance, where stable and self-amplifying small-RNA systems may provide a mechanistic basis for the long-term transmission of environmentally induced phenotypic variation.

#### 3.1.2. Transgenerational piRNA Inheritance in *C. elegans*

Studies in *Caenorhabditis elegans*, originally published in 2012, have provided some of the strongest experimental evidence that small RNAs can initiate and maintain transgenerational epigenetic inheritance programs across multiple generations [26,27,69,70,71,72,73,74,75,76,77]. In this nematode system, piRNAs function not only as regulators of transposable element silencing but also as initiators of stable gene-silencing pathways that can persist long after the original trigger has disappeared [26,27,72]. These findings have established *C. elegans* as a central model organism for understanding how environmentally responsive RNA signals may become heritable epigenetic information.

In *C. elegans*, piRNAs—also referred to as 21U-RNAs because of their characteristic length and 5′ uridine bias—associate with the PIWI-family protein PRG-1 to recognize foreign or aberrant nucleic acid sequences [78,79]. Upon target recognition, PRG-1/piRNA complexes initiate silencing by recruiting RNA-dependent RNA polymerases (RdRPs), which generate large populations of secondary small interfering RNAs known as 22G-RNAs [80,81]. These secondary RNAs amplify the original silencing signal and are associated with downstream Argonaute proteins that mediate robust transcriptional and post-transcriptional repression of target genes. Importantly, this amplification process allows relatively small numbers of initiating piRNAs to generate durable and self-reinforcing silencing responses.

One of the most remarkable discoveries in this system is that piRNA-triggered silencing can persist for many generations after the initial piRNA trigger is lost [26,27,72]. This phenomenon, often referred to as RNA-induced epigenetic silencing (RNAe), demonstrates that epigenetic states initiated by small RNAs can become self-sustaining through interactions between secondary small-RNA pathways and chromatin regulatory mechanisms. Maintenance of these inherited silencing states involves nuclear Argonaute proteins and deposition of repressive chromatin marks, including histone H3 lysine 9 methylation (H3K9me), which stabilizes transcriptional repression across generations [26,27,72].

These transgenerational inheritance systems also exhibit properties consistent with epigenetic memory. Once established, silenced loci can remain repressed for more than twenty generations in some experimental contexts, even in the absence of the original environmental or genetic trigger [26,27,72]. Conversely, *C. elegans* also possesses active mechanisms that prevent uncontrolled propagation of epigenetic silencing, including pathways that reset or limit inherited small-RNA signals over time [69]. These balancing mechanisms suggest that transgenerational epigenetic inheritance is dynamically regulated rather than permanently fixed.

Importantly, the *C. elegans* system provides a mechanistic framework that may help explain how environmentally induced small-RNA signals persist across generations. Unlike many other small-RNA classes, piRNA-associated pathways in *C. elegans* possess intrinsic amplification systems through RdRP-dependent secondary RNA production, allowing silencing signals to be continuously reinforced [82]. This feature is particularly relevant when considering broader models of transgenerational inheritance because it addresses a major conceptual challenge in the field: how transient environmental signals can generate stable heritable effects that persist beyond the initial exposure.

The *C. elegans* literature also highlights important parallels between transposon defense and epigenetic inheritance. Many piRNA pathways evolved initially to silence foreign genetic elements such as transposons and viruses, yet these same pathways can also regulate endogenous genes and heritable chromatin states [83]. This evolutionary connection suggests that mechanisms originally developed for genome defense may have been co-opted for broader functions in environmental adaptation and transgenerational phenotypic regulation.

Together, these findings establish that piRNA-mediated pathways in *C. elegans* can initiate, amplify, and maintain heritable epigenetic states across generations. These studies provide an important conceptual and mechanistic foundation for models proposing that small RNAs—including piRNAs associated with stress responses or immune-cell signaling—may contribute to transgenerational inheritance in more complex organisms.

#### 3.1.3. Small-RNA Inheritance Systems in Plants and Other Eukaryotes

**Figure 3:**
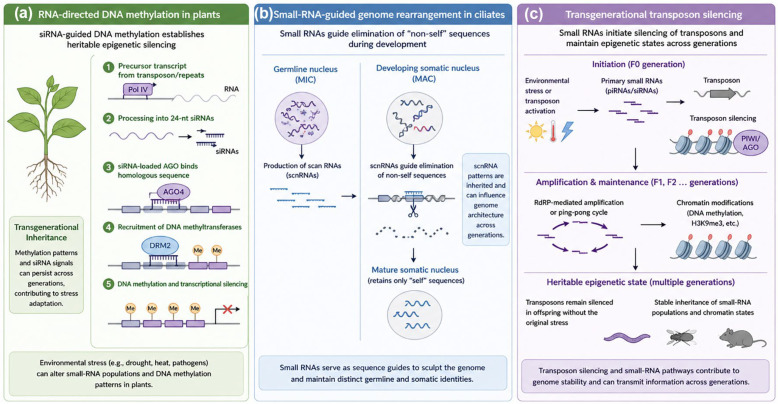
Small-RNA inheritance systems in plants and other eukaryotes. This figure summarizes evolutionarily conserved small-RNA-guided mechanisms that contribute to transgenerational epigenetic inheritance across diverse eukaryotic systems. (**a**) RNA-directed DNA methylation (RdDM) in plants. In plants, transposon- or repeat-derived precursor RNAs are processed into 24-nt small interfering RNAs (siRNAs), which are associated with Argonaute proteins and guide DNA methyltransferases to homologous genomic loci. This process establishes transcriptionally repressive DNA methylation patterns that silence transposable elements and repetitive DNA. These epigenetic modifications can persist across generations and may be altered by environmental stressors such as drought, heat, or pathogen exposure. Argonaut 4 (AGO4) is a plant PIWI family member. Domanains Rearranged Methyltransferase 2 is a plant DNA methyltransferase. Polymerase IV (Pol IV) is a plant RNA polymerase. (**b**) Small-RNA-guided genome rearrangement in ciliates. In ciliates such as *Tetrahymena* and *Paramecium*, small RNAs known as scan RNAs (scnRNAs) are generated from the germline micronucleus and guide elimination of “non-self” DNA sequences during development of the somatic macronucleus. These pathways enable selective genome remodeling and maintenance of distinct germline and somatic genome architectures across generations. Micronucleus (MIC) and Macronucleus (MAC) are germline and somatic nuclei. (**c**) Transgenerational transposon silencing mechanisms in animals and other eukaryotes. Environmental stress or transposon activation induces production of small RNAs, including piRNAs and siRNAs, which initiate silencing of transposable elements. Amplification systems such as RNA-dependent RNA polymerase (RdRP) pathways or the piRNA ping-pong cycle reinforce these silencing signals. Small-RNA-guided chromatin modifications, including DNA methylation and H3K9me3 deposition, stabilize heritable epigenetic states that can persist across multiple generations after the original environmental trigger has disappeared. Together, these systems demonstrate that small-RNA-mediated genome regulation is phylogenetically widespread and supports the broader concept that RNA-guided epigenetic pathways can transmit information about environmental stress and genome state across generations.

Although canonical PIWI–piRNA pathways are best characterized in animals, a growing body of evidence from plants and other eukaryotic systems demonstrates that small-RNA-guided epigenetic inheritance is evolutionarily widespread [31,84,85,86,87,88,89,90,91,92,93,94,95,96] (Figure 3). These systems provide important conceptual support for the idea that organisms can transmit environmentally responsive epigenetic information across generations through RNA-mediated mechanisms. While the molecular components differ among taxa, many of these pathways converge functionally on the regulation of transposable elements, chromatin organization, and heritable genome stability.

In plants, one of the best-characterized small-RNA inheritance systems is RNA-directed DNA methylation (RdDM), a pathway in which small interfering RNAs (siRNAs) guide DNA methylation and transcriptional silencing of repetitive elements and transposons [88,90,93,95]. In this process, specialized plant RNA polymerases generate precursor transcripts that are processed into 24-nucleotide siRNAs, which are associated with Argonaute proteins and recruit DNA methyltransferases to homologous genomic regions [48]. This targeting results in de novo DNA methylation and stable transcriptional repression of transposable elements. Importantly, these methylation patterns can persist across generations, allowing environmentally induced epigenetic states to be inherited by offspring [88,90,93,95]. Environmental stressors such as temperature fluctuations, pathogen exposure, and nutrient stress have been shown to alter small-RNA populations and DNA methylation patterns in plants, further supporting the idea that RNA-mediated epigenetic responses can participate in adaptive stress responses [85,87,95,97,98]. Although plants lack canonical piRNAs, the RdDM system provides a compelling parallel to animal piRNA pathways because both systems use small RNAs to guide heritable epigenetic silencing of repetitive genomic elements.

Additional support for RNA-mediated inheritance mechanisms comes from unicellular eukaryotes, particularly ciliates such as *Tetrahymena*, *Paramecium*, and *Oxytricha* [31,32,33]. These organisms possess highly unusual nuclear architectures involving separate germline and somatic nuclei, and they use small-RNA-guided mechanisms to direct large-scale genome rearrangements during development. In ciliates, small RNAs known as scan RNAs (scnRNAs) are generated from germline genomes and used to identify DNA sequences that should be eliminated from the developing somatic nucleus [32,33]. Through this process, small RNAs effectively distinguish “self” from “non-self” genomic regions and guide selective removal or retention of DNA sequences during nuclear differentiation. These RNA-guided genome remodeling pathways demonstrate that small RNAs can regulate not only gene expression but also large-scale structural organization of genomes across generations.

Small-RNA-mediated transgenerational silencing mechanisms have also been described in fungi, algae, and other lower eukaryotes [99,100]. In many of these systems, RNA-dependent RNA polymerases amplify small-RNA signals through feedback loops that reinforce gene silencing and maintain epigenetic states over time [101]. These amplification systems are conceptually important because they provide mechanisms by which transient environmental signals can become stable and self-reinforcing across multiple generations. Such features parallel the ping-pong amplification pathways observed in animal piRNA systems and address one of the major conceptual challenges in transgenerational epigenetic inheritance: how ephemeral environmental exposures can generate durable biological effects.

A recurring theme across these diverse organisms is the close relationship between small-RNA pathways and transposable element regulation. Transposons are major drivers of genomic instability and evolutionary innovation, and many small-RNA inheritance systems appear to have evolved initially as defense mechanisms against transposable elements and foreign nucleic acids [102,103]. Over evolutionary time, these genome-defense pathways may have been co-opted for broader functions in developmental regulation, environmental adaptation, and epigenetic inheritance. This evolutionary connection between genome defense and heritable epigenetic regulation is particularly relevant to emerging models proposing that stress-responsive small RNAs may contribute to environmentally induced phenotypic variation across generations.

Together, studies in plants, ciliates, and other eukaryotes demonstrate that small-RNA-guided inheritance systems are phylogenetically widespread and mechanistically diverse. These systems support the broader concept that RNA-mediated epigenetic pathways can transmit information about genome state and environmental conditions across generations. Such findings provide an important evolutionary framework for understanding how piRNA-associated pathways in animals, potentially including macrophage-associated piRNA signaling mechanisms, may contribute to transgenerational inheritance in more complex organisms.

### 3.2. Environmental Stress, Hsp90, and Transposon Activation

#### 3.2.1. Hsp90 as a Capacitor for Phenotypic Variation

The molecular chaperone heat-shock protein 90 (Hsp90) has emerged as one of the most important regulators linking environmental stress responses to phenotypic variation and developmental stability [54,55,56,104,105,106,107,108,109,110,111,112,113,114,115,116,117,118,119,120]. Hsp90 is a highly conserved ATP-dependent chaperone that stabilizes and assists in the folding of a wide range of client proteins, many of which are central regulators of signal transduction, development, chromatin regulation, and cellular stress responses [56,114]. Under normal physiological conditions, Hsp90 functions as a buffering system that stabilizes metastable proteins and suppresses the phenotypic consequences of underlying genetic and epigenetic variation. This buffering capacity led to the proposal that Hsp90 acts as a “capacitor” for morphological evolution by concealing cryptic variation until environmental stress disrupts its function [56,114].

**Figure 4:**
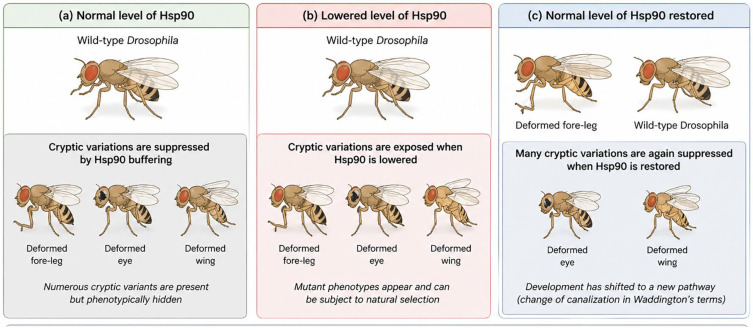
Exposing cryptic genetic variation: the Hsp90 capacitor function in *Drosophila*. (**a**) When heat-shock protein Hsp90 is expressed at normal levels, wild-type *Drosophila* develops with a normal phenotype (top panel). Under these conditions, numerous cryptic genetic and epigenetic variants remain phenotypically suppressed by the buffering activity of Hsp90 and are therefore not expressed. (**b**) When Hsp90 levels are reduced by genetic disruption, pharmacological inhibition (e.g., geldanamycin), or environmental stress such as heat shock, the buffering capacity of Hsp90 is compromised. As a result, previously hidden cryptic variation becomes phenotypically expressed, leading to diverse developmental abnormalities including deformed forelegs, eye defects, and wing abnormalities. These newly exposed phenotypes can subsequently be subjected to natural or artificial selection. (**c**) After several generations of selection for a specific phenotype (illustrated here as a deformed foreleg phenotype), Hsp90 levels are restored to normal. Although many cryptic variants are once again suppressed, selected phenotypes can persist despite restoration of normal Hsp90 function, suggesting that developmental pathways have shifted into a new stable state of canalization in the sense originally proposed by Waddington. This figure is redrawn from Ann McLaren’s review [1].

The concept of Hsp90 as a capacitor for phenotypic variation was first strongly supported by experiments demonstrating that partial inhibition of Hsp90 in *Drosophila melanogaster* produced diverse morphological abnormalities, including altered eye structures, wing defects, and ectopic developmental phenotypes (Figure 4) [1,56,113,114,121]. Similar findings were later reported in plants, fungi, fish, and mammalian systems, suggesting that Hsp90-dependent buffering mechanisms are broadly conserved across eukaryotes [106,110,119]. Importantly, many of these stress-induced phenotypes could be selected over multiple generations, implying that Hsp90 suppression revealed previously hidden developmental variation that could become evolutionarily relevant under conditions of environmental challenge.

One explanation for these observations is that Hsp90 stabilizes numerous developmental regulators that would otherwise be functionally unstable. These include proteins involved in major signaling pathways such as MAPK/ERK, PI3K/AKT, steroid hormone signaling, and JAK/STAT pathways [122]. Under non-stress conditions, Hsp90 maintains proper folding and function of these regulatory proteins, thereby preserving developmental robustness. However, during environmental stress—including heat shock, oxidative stress, toxicant exposure, or nutrient deprivation—Hsp90 becomes increasingly occupied by damaged or misfolded proteins [122]. As Hsp90 buffering capacity becomes overwhelmed, unstable client proteins may lose function or become dysregulated, exposing latent phenotypic variation that was previously suppressed.

Beyond its role in buffering developmental signaling pathways, Hsp90 also interacts extensively with epigenetic regulatory systems [54,55,56,104,106,108,112,119]. Studies have demonstrated that Hsp90 associates with chromatin remodeling complexes, histone methyltransferases, histone deacetylases, and DNA methyltransferases [56,106,119]. These interactions suggest that Hsp90 contributes not only to protein stability but also to maintenance of epigenetic states and chromatin organization. Of particular importance is the interaction between Hsp90 and PIWI-family proteins involved in piRNA-mediated transposon repression [53,123]. Hsp90 facilitates proper loading, stabilization, and activation of PIWI proteins, and disruption of Hsp90 function impairs piRNA pathways and transposon silencing [123]. Consequently, environmental stress that compromises Hsp90 activity may simultaneously destabilize both developmental signaling networks and epigenetic genome defense systems.

This connection between Hsp90 and epigenetic regulation has led to the hypothesis that Hsp90 buffers contain not only cryptic genetic variation but also cryptic epigenetic variation [54,55,121]. Supporting this idea, inhibition of Hsp90 can induce activation of transposable elements, chromatin instability, and altered gene-expression states that persist beyond the original stress exposure [56,114]. Such observations are particularly relevant to models of transgenerational epigenetic inheritance because activation of transposons and associated small-RNA responses may generate heritable epigenetic signals capable of influencing future generations.

Studies from Drosophila systems provide particularly striking examples of this phenomenon. In the ectopic large bristle outgrowth (ELBO) model, discussed in a later section, inhibition of Hsp90 using the geldanamycin compound induced ectopic appendage-like structures in the eye and increased phenotypic variability across generations [53,55,56]. The incomplete penetrance and rapid reversibility of these phenotypes suggested that they were not solely driven by stable genetic mutations but instead involved environmentally responsive epigenetic mechanisms. Such findings support the broader hypothesis that Hsp90 functions at the interface between environmental stress responses, developmental regulation, and epigenetic inheritance.

Together, these studies establish Hsp90 as a central regulator of developmental robustness and stress-responsive phenotypic variation. By buffering both signaling pathways and epigenetic regulatory systems, Hsp90 may act as a critical mediator linking environmental stress to the release of hidden developmental and epigenetic variation. These functions make Hsp90 particularly relevant to emerging models proposing that stress-induced activation of small-RNA pathways, including piRNA-mediated mechanisms, contributes to environmentally induced transgenerational inheritance.

#### 3.2.2. Environmental Stress and Small-RNA Responses

**Figure 5:**
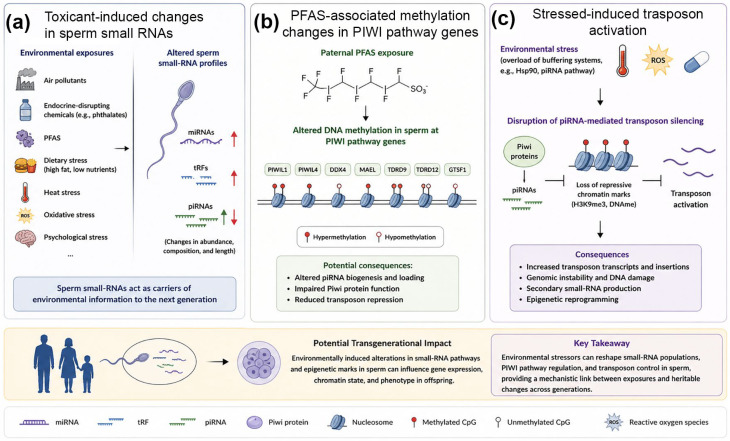
Environmental stress alters small-RNA pathways and sperm epigenetic states. This graphic abstract summarizes how environmental stressors disrupt small-RNA pathways, transposon repression systems, and germline epigenetic regulation, potentially contributing to transgenerational epigenetic inheritance. (**a**) Toxicant-induced changes in sperm small RNAs. Multiple environmental stressors—including air pollutants, endocrine-disrupting chemicals such as phthalates, PFAS compounds, dietary stress, heat stress, oxidative stress, and psychological stress—alter sperm-associated small-RNA populations. These changes affect multiple classes of small RNAs, including microRNAs (miRNAs), tRNA-derived fragments (tRFs), and piRNAs, resulting in altered sperm RNA composition and potential transmission of environmentally responsive signals to offspring. Perfluoroalkysubstances (PFAS), miRNAs (microRNAs), and tRNA fragments (tRFs) are indicated. (**b**) PFAS-associated methylation changes in PIWI pathway genes. Paternal exposure to PFAS and related environmental toxicants is associated with altered DNA methylation patterns in sperm at genes involved in piRNA biogenesis and transposon repression pathways, including PIWIL1, PIWIL4, DEAD-box helicase 4, also known as Vasa (DDX4), Maelstrom (MAEL), Tudor domain-containing protein 9 and 12 (TDRD9, TDRD12), and Gametocyte-Specific Factor 1 (GTSF1). These epigenetic alterations may impair piRNA pathway function, disrupt PIWI protein activity, and weaken transposon repression systems in germ cells. (**c**) Stress-induced transposon activation. Environmental stress can overload cellular buffering systems, including Hsp90-dependent regulatory pathways and piRNA-mediated genome-defense systems. Disruption of PIWI/piRNA pathways leads to loss of repressive chromatin marks such as H3K9me3 and DNA methylation, resulting in transposon activation, increased transposon-derived transcripts, genomic instability, DNA damage, and secondary small-RNA production. These events may contribute to epigenetic reprogramming and environmentally induced phenotypic variation. Together, these pathways illustrate how environmental stressors can reshape small-RNA populations, alter epigenetic regulation in sperm, and destabilize transposon repression systems. Such environmentally induced changes may provide a mechanistic link between environmental exposures and transgenerational epigenetic inheritance.

A substantial body of evidence now indicates that environmental stressors can alter small-RNA pathways [124,125,126,127], epigenetic regulatory systems [128,129,130,131], and germline chromatin states [7,78,132,133,134,135,136,137,138,139,140,141] (Figure 5). Environmental toxicants, heat stress, oxidative stress, endocrine-disrupting chemicals, and inflammatory exposures have all been associated with changes in small-RNA populations in germ cells and sperm, raising the possibility that environmentally induced alterations in RNA-mediated signaling pathways may contribute to transgenerational phenotypic inheritance [6,7,8,11,16,18,34,35,36,83,135,140]. These observations are particularly important because they provide potential mechanistic links between somatic stress responses and heritable epigenetic changes transmitted to offspring.

One of the strongest areas of evidence comes from studies demonstrating that environmental exposures alter small-RNA profiles in sperm. Multiple toxicants, including endocrine-disrupting compounds, air pollutants, heavy metals, and dietary stressors, have been shown to induce significant changes in sperm-associated non-coding RNAs [8,11,16,18,35,83]. These altered RNA populations include miRNAs, tRNA-derived fragments (tRFs), and piRNAs. For example, paternal exposure to environmental toxicants such as phthalates has been associated with substantial remodeling of sperm small-RNA populations, including increased abundance of specific piRNA subclasses [60,61,83]. Similar findings have been observed following exposure to cigarette smoke, high-fat diets, and psychological stress paradigms, suggesting that sperm small-RNA populations are highly responsive to environmental conditions [142,143].

Although many studies have focused primarily on miRNAs and tRFs, relatively fewer investigations have specifically characterized environmentally responsive piRNA populations. This relative lack of attention may partially reflect technical limitations, since piRNAs require specialized sequencing and enrichment approaches due to their unique biochemical properties, including the 3′ terminal 2′-O-methyl modification [24,123,144]. Nevertheless, available evidence suggests that piRNA pathways are highly sensitive to environmental perturbation. Because piRNAs function centrally in transposon repression and chromatin regulation, environmentally induced disruption of piRNA populations could have particularly important consequences for genome stability and epigenetic inheritance.

Environmental stressors have also been linked to epigenetic alterations in genes associated with PIWI pathways. Recent studies examining paternal exposure to mixtures of per- and polyfluoroalkyl substances (PFASs) demonstrated altered DNA methylation patterns in sperm at multiple genes involved in piRNA biogenesis and transposon repression [36,145]. These included methylation changes in PIWI-family genes such as Piwil1 and Piwil4, as well as associated pathway components including DDX4, MAEL, TDRD9, TDRD12, and GTSF1 [36,145]. Such findings are particularly significant because these genes are essential regulators of piRNA biogenesis, transposon silencing, and germline genome stability. Altered methylation of these loci may therefore influence both piRNA pathway activity and susceptibility to transposon activation in subsequent generations.

Additional support for environmentally induced epigenetic instability comes from studies demonstrating altered sperm methylation patterns associated with reproductive dysfunction and infertility. For example, sperm from men with prolonged time-to-pregnancy intervals have been reported to exhibit significant changes in DNA methylation at loci associated with developmental regulation and epigenetic pathways [146,147,148,149,150,151,152,153,154,155,156]. Although the mechanistic relationship between these methylation changes and small-RNA pathways remains incompletely understood, these findings suggest that environmental and physiological stressors can induce coordinated alterations in sperm epigenetic architecture.

A major consequence of stress-induced disruption of small-RNA pathways is activation of transposable elements. Under normal conditions, transposons are tightly repressed through coordinated actions of piRNAs, DNA methylation, and heterochromatin formation [22,59,60,65,67,92,157,158,159,160]. However, environmental stress—including heat shock, oxidative stress, aging, toxicant exposure, and Hsp90 inhibition—has been shown to destabilize these silencing systems and promote transposon expression [157]. Increased transposon activity can generate genomic instability through insertional mutagenesis, DNA breaks, and chromosomal rearrangements. Importantly, transposon activation also produces abundant transposon-derived RNA substrates that can feed into small-RNA amplification pathways, including piRNA ping-pong cycles and secondary RNA production systems [22,59,60,67,157,159,160].

This relationship between environmental stress and transposon activation has important implications for epigenetic inheritance. Because transposons are major targets of small-RNA-guided chromatin regulation, stress-induced transposon expression may trigger compensatory small-RNA responses that reshape epigenetic states [161,162]. In some contexts, these responses may persist after the original stress exposure has ended, potentially contributing to stable transgenerational epigenetic changes. Such mechanisms provide a plausible biological framework through which environmental stress could generate heritable alterations in gene regulation without requiring permanent DNA sequence mutations.

These observations are particularly relevant to models proposing that immune cells and stress-responsive pathways contribute to epigenetic inheritance. Environmental stress that disrupts Hsp90 buffering and piRNA-mediated transposon repression may simultaneously generate damaged cells, inflammatory signaling, and altered small-RNA populations. In this context, macrophages and macrophage-derived extracellular vesicles may represent potential intermediaries capable of integrating stress-induced small-RNA signals and distributing them systemically, including germline tissues.

Together, current evidence demonstrates that environmental stressors can profoundly alter small-RNA populations, transposon regulation, and germline epigenetic states. Toxicant-induced changes in sperm small RNAs, methylation alterations in PIWI pathway genes, and stress-induced transposon activation collectively support the broader concept that environmental exposures can reshape epigenetic regulatory systems in ways that may influence future generations. These findings provide an important mechanistic foundation for emerging models linking environmental stress, small-RNA biology, and transgenerational epigenetic inheritance.

### 3.3. Macrophages as Regulators of Tissue Remodeling and Stress Responses

#### 3.3.1. Evolutionary Origins of Macrophage-Like Cells

**Figure 6:**
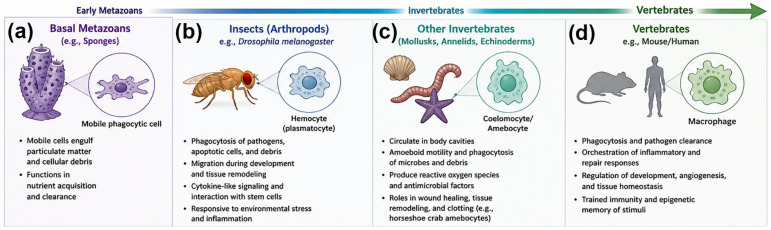
Evolutionary origins and conserved functions of macrophage-like cells across metazoans. This figure summarizes the evolutionary conservation of macrophage-like phagocytic cells from basal metazoans to vertebrates and highlights their shared roles in immunity, tissue remodeling, environmental sensing, and intercellular communication. (**a**) Basal metazoans. Primitive multicellular organisms such as sponges possess mobile phagocytic cells capable of engulfing particulate matter and cellular debris. These early phagocytic cells likely evolved initially for nutrient acquisition and tissue maintenance before becoming specialized immune cells. (**b**) Insects and arthropods. In insects such as *Drosophila* melanogaster, macrophage-like hemocytes (plasmatocytes) perform many functions analogous to vertebrate macrophages, including phagocytosis of pathogens and apoptotic cells, developmental tissue remodeling, cytokine-like signaling, and responses to environmental stress and inflammation. (**c**) Other invertebrates. Mollusks, annelids, echinoderms, and other invertebrates contain macrophage-like coelomocytes or amebocytes that circulate through body cavities and participate in phagocytosis, wound healing, antimicrobial defense, reactive oxygen species production, and tissue remodeling. (**d**) Vertebrates. Vertebrate macrophages perform highly specialized functions in innate immunity, inflammatory regulation, tissue repair, angiogenesis, developmental morphogenesis, and maintenance of tissue homeostasis. These cells also exhibit forms of trained immunity and epigenetic memory following environmental exposures.

Phagocytic immune cells are among the most evolutionarily ancient cell types in multicellular organisms and are thought to have emerged early in metazoan evolution as fundamental regulators of defense, tissue remodeling, and cellular homeostasis (Figure 6) [163,164]. Although vertebrate macrophages are highly specialized components of the innate immune system, macrophage-like cells with remarkably similar functional properties are found throughout the animal kingdom, including in insects, mollusks, annelids, echinoderms, and primitive metazoans [163,164,165]. The widespread distribution of these phagocytic cells suggests that core macrophage functions evolved long before the emergence of adaptive immunity and may represent deeply conserved biological programs associated with environmental sensing, tissue surveillance, and removal of damaged cellular material.

In insects such as *Drosophila melanogaster*, macrophage-like immune cells known as hemocytes perform many functions analogous to vertebrate macrophages [166,167,168,169,170,171,172]. The major hemocyte subtype in Drosophila larvae, known as plasmatocytes, exhibits strong phagocytic activity and participates in host defense, apoptotic cell clearance, extracellular matrix remodeling, and developmental tissue sculpting [166,171,172]. During embryogenesis and larval development, hemocytes migrate extensively through tissues and engulf apoptotic cells generated during morphogenesis. These cells also produce cytokine-like signaling molecules and interact with stem and progenitor cell populations involved in tissue regeneration and wound repair [166,171,172]. Importantly, Drosophila hemocytes are highly responsive to environmental stress and inflammatory stimuli, making them particularly relevant to models linking stress responses to developmental plasticity and epigenetic regulation.

Macrophage-like phagocytic cells are also widely distributed among non-arthropod invertebrates. In mollusks, annelids, and echinoderms, phagocytic immune cells known as coelomocytes or amebocytes circulate through body cavities and participate in pathogen defense, wound healing, and tissue remodeling [173,174,175,176]. These cells display amoeboid motility, actively engulf foreign particles and dying cells, and produce reactive oxygen species and inflammatory signaling molecules like vertebrate macrophages. In horseshoe crabs and other arthropods, amebocytes also contribute to clotting and antimicrobial defense systems, further illustrating the multifunctional roles of ancient phagocytic cell types [173,174,175,176,177].

Even basal metazoans such as sponges possess mobile phagocytic cells capable of engulfing particulate material and clearing cellular debris [178]. These observations suggest that phagocytosis evolved initially as a mechanism for nutrient acquisition and cellular waste management before later becoming integrated into specialized immune-defense systems. Over evolutionary time, these primitive phagocytic programs may have diversified into the complex macrophage and innate immune systems observed in modern vertebrates.

One of the most striking features of macrophage-like cells across species is the strong conservation of their core cellular functions. Across metazoans, these cells exhibit conserved abilities to migrate toward damaged tissues, recognize stressed or apoptotic cells, engulf cellular debris through phagocytosis, remodel extracellular environments, and coordinate inflammatory signaling responses [173,174,175,176,177]. These highly conserved properties suggest that phagocytic cells serve as central integrators of tissue stress and environmental information. Such functions position macrophage-like cells as plausible intermediaries between environmental perturbations and long-term physiological adaptation.

In addition to their roles in immunity, macrophage-like cells also contribute extensively to developmental morphogenesis and tissue patterning. In vertebrates, macrophages regulate branching morphogenesis, angiogenesis, adipose tissue remodeling, and stem-cell niche maintenance [5,179,180]. Similarly, Drosophila hemocytes contribute to tissue remodeling during metamorphosis and influence developmental signaling environments within damaged tissues [166,171,172]. These developmental functions are particularly relevant to hypotheses proposing that macrophage-like cells may influence environmentally responsive phenotypic variation and morphological plasticity.

Recent evidence further suggests that macrophages possess forms of “innate immune memory” or trained immunity in which prior environmental exposures alter future macrophage responses through epigenetic remodeling pathways [181,182]. Such observations indicate that macrophages are not merely passive responders to tissue damage but can maintain long-term molecular memory of environmental stimuli. Although most studies of trained immunity focus on inflammatory responses, these findings raise the intriguing possibility that macrophage-associated epigenetic states or small-RNA pathways could participate in broader forms of biological memory, including potentially heritable responses to environmental stress.

From an evolutionary perspective, the ancient origins and conserved biological functions of macrophage-like cells make them compelling candidates for mediators of environmentally responsive signaling systems. Their abilities to integrate stress signals, migrate systemically, engulf damaged cellular material, and release extracellular vesicles containing regulatory RNAs suggest that they may serve as mobile communication hubs linking tissue stress responses to systemic physiological regulation. These properties form an important conceptual foundation for the macrophage-mediated morphological (M3) model proposed in this review, in which macrophage-like cells may participate in transmission of environmentally induced small-RNA signals that influence developmental and potentially transgenerational phenotypes.

#### 3.3.2. Macrophage Polarization and Functional Plasticity

**Figure 7:**
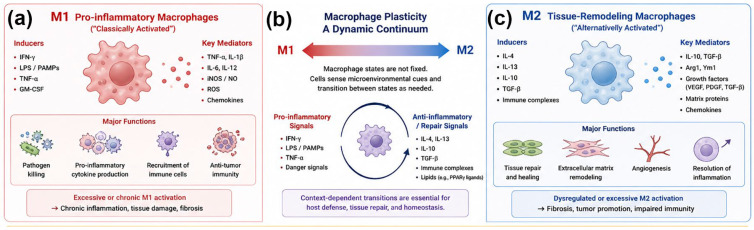
Macrophage polarization and functional plasticity in inflammation, tissue remodeling, and developmental signaling. This figure summarizes the dynamic functional states of macrophages and their roles in immune regulation, tissue remodeling, developmental signaling, and environmentally responsive plasticity. (**a**) M1 pro-inflammatory macrophages. Classically activated M1 macrophages are induced by inflammatory stimuli, including interferon-γ (IFN-γ), lipopolysaccharide (LPS), pathogen-associated molecular patterns (PAMPs), and tumor necrosis factor-α (TNF-α). These macrophages produce pro-inflammatory mediators, including TNF-α, IL-1β, IL-6, inducible nitric oxide synthases (iNOS), nitric oxide, reactive oxygen species (ROS), and chemokines. M1 macrophages function in pathogen killing, inflammatory cytokine production, recruitment of immune cells, and antitumor immunity. Chronic or excessive M1 activation can contribute to persistent inflammation, fibrosis, and tissue damage. (**b**) M2 tissue-remodeling macrophages. Alternatively activated M2 macrophages are induced by signals such as IL-4, IL-13, IL-10, TGF-β, and anti-inflammatory tissue environments. M2 macrophages produce anti-inflammatory cytokines, extracellular matrix proteins, angiogenic growth factors, and tissue-remodeling mediators that support wound healing, extracellular matrix remodeling, angiogenesis, stem-cell regulation, and resolution of inflammation. Dysregulated M2 activity may contribute to fibrosis, impaired immune responses, and tumor progression. (**c**) Macrophage plasticity and dynamic transitions. Macrophage activation states exist along a dynamic continuum rather than as fixed cell types. Macrophages continuously integrate environmental, inflammatory, and developmental signals and can transition between M1 and M2 states depending on tissue context and physiological demands. These transitions are critical for balancing host defense, tissue repair, and maintenance of homeostasis.

Macrophages are highly plastic immune cells capable of adopting diverse functional states in response to environmental, developmental, and inflammatory signals (Figure 7) [183,184]. Rather than existing as a single uniform cell type, macrophages dynamically alter their transcriptional programs, metabolic states, cytokine profiles, and tissue functions depending on local microenvironmental cues. This remarkable functional flexibility allows macrophages to participate in a wide range of biological processes, including host defense, tissue repair, developmental morphogenesis, stem-cell regulation, and maintenance of tissue homeostasis [183,184]. Among the best-characterized macrophage activation states are the classically activated M1 phenotype and the alternatively activated M2 phenotype, although contemporary studies increasingly recognize macrophage activation as a continuum rather than a strict binary classification [46,183].

M1 macrophages are generally associated with pro-inflammatory and antimicrobial responses [184,185]. These cells are activated by stimuli such as interferon-γ (IFN-γ), lipopolysaccharide (LPS), pathogen-associated molecular patterns, and inflammatory cytokines. Once activated, M1 macrophages produce high levels of pro-inflammatory mediators, including tumor necrosis factor-α (TNF-α), interleukin-1β (IL-1β), interleukin-6 (IL-6), nitric oxide, and reactive oxygen species [184,185]. These responses are critical for pathogen clearance and early inflammatory defense mechanisms. M1 macrophages also participate in antitumor immunity by promoting inflammatory microenvironments that suppress tumor growth and recruit additional immune effector cells [184,185]. However, prolonged or excessive M1 activation can contribute to chronic inflammation, fibrosis, and tissue damage.

In contrast, M2 macrophages are generally associated with tissue repair, wound healing, extracellular matrix remodeling, and resolution of inflammation [44,186]. M2 polarization is stimulated by cytokines such as IL-4 and IL-13 as well as anti-inflammatory tissue environments. These macrophages produce growth factors, anti-inflammatory cytokines, and extracellular matrix components that support tissue regeneration and remodeling [44,186]. M2 macrophages also contribute to angiogenesis, stem-cell niche maintenance, and restoration of tissue homeostasis following injury. In many developmental contexts, M2-like macrophages participate in morphogenetic remodeling by removing apoptotic cells and coordinating regenerative signaling programs [44,186].

Importantly, macrophage polarization states are not fixed and can dynamically shift in response to changing physiological conditions. In many biological systems, transitions between M1 and M2 states are essential for normal tissue remodeling processes [184,187,188]. For example, during wound healing, an initial M1 inflammatory response is often followed by a transition toward M2 repair-associated macrophages that promote tissue regeneration and suppress excessive inflammation. Dysregulation of these transitions can contribute to pathological outcomes, including chronic inflammation, fibrosis, impaired wound healing, and tumor progression [189,190].

Recent studies have also revealed important links between macrophage polarization and small-RNA regulatory pathways, including piRNA-associated signaling systems [47,48,191]. Distinct piRNA expression profiles have been identified in M1 and M2 macrophages, suggesting that small-RNA-mediated regulatory mechanisms may contribute to macrophage functional specialization. Certain piRNAs appear to promote pro-inflammatory M1 signaling pathways through regulation of TNF-α expression, whereas others influence M2-associated pathways involving HIF-1α signaling and tissue remodeling responses [39,40]. These observations suggest that macrophage polarization states may be partially stabilized or regulated through epigenetic and small-RNA-mediated mechanisms.

**Figure 8:**
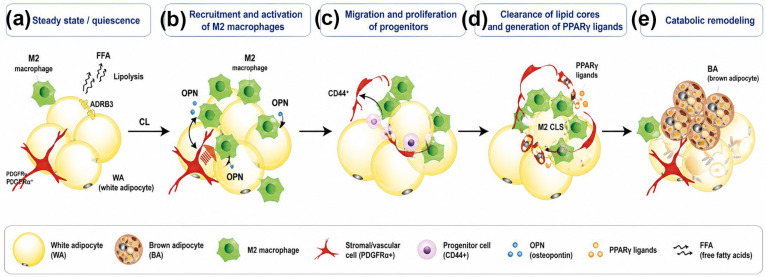
Macrophage-mediated adipose tissue remodeling during white-to-brown adipocyte conversion. This figure summarizes a proposed sequence of macrophage-dependent remodeling events involved in environmentally induced conversion of white adipose tissue (WAT) into thermogenic brown adipose tissue (BAT) during cold adaptation. The model is adapted from adipose tissue remodeling studies demonstrating coordinated interactions between adipocytes, macrophages, stromal cells, and progenitor cells during tissue remodeling and thermogenic reprogramming. (**a**) Steady-state quiescence. Under basal conditions, white adipocytes (WA) are maintained within adipose tissue together with resident M2-like macrophages and stromal/vascular progenitor cells expressing platelet-derived growth factor receptor-α (PDGFRα). Adrenergic signaling through ADRB3 receptors regulates adipocyte metabolic activity and lipolysis. (**b**) Recruitment and activation of M2 macrophages. Environmental stress, such as cold exposure, lipolysis and release of free fatty acids (FFAs), leading to recruitment and activation of macrophages within adipose tissue. Osteopontin (OPN) signaling contributes to macrophage recruitment and local inflammatory remodeling responses. (**c**) Migration and proliferation of progenitor cells. Activated macrophages and stromal signals promote migration and proliferation of progenitor cells expressing CD44 and related stem/progenitor markers. Macrophages coordinate local remodeling environments by producing cytokines, growth factors, and extracellular matrix remodeling signals. (**d**) Clearance of lipid cores and generation of PPARγ ligands. Macrophages form crown-like structures (CLS) surrounding damaged or dying adipocytes and phagocytose lipid-rich cellular debris. This remodeling process contributes to generation of PPARγ ligands and activation of signaling pathways involved in adipocyte differentiation and metabolic reprogramming. (**e**) Catabolic remodeling and brown adipocyte formation. Remodeling signals generated during macrophage-mediated tissue restructuring promote differentiation of thermogenic brown adipocytes (BA), characterized by increased mitochondrial density and enhanced energy expenditure. This adaptive remodeling response improves thermogenesis and physiological adaptation to cold stress. Together, these findings illustrate how macrophages function not only as inflammatory scavenger cells but also as active regulators of tissue remodeling, progenitor-cell activation, and environmentally responsive developmental plasticity. These remodeling functions support the broader hypothesis proposed in the macrophage-mediated morphological (M3) model that macrophages may coordinate adaptive tissue responses through signaling pathways that integrate environmental stress, developmental remodeling, and epigenetic regulation. Figure adapted from [5].

One particularly relevant example involves remodeling of adipose tissue during cold-induced browning responses. Studies from the Granneman laboratory demonstrated that macrophages participate directly in conversion of white adipose tissue to thermogenic brown adipose tissue during cold stress (Figure 8) [5]. In this process, macrophages engulf dying adipocytes and produce signaling molecules that influence adipocyte progenitor differentiation and tissue remodeling. Importantly, shifts between inflammatory and repair-associated macrophage states appear to regulate these remodeling programs. Such findings support the broader concept that macrophages can act not only as scavenger cells but also as active coordinators of developmental and regenerative plasticity.

These developmental and remodeling functions are highly relevant to hypotheses proposing that macrophages contribute to environmentally responsive phenotypic variation. Because macrophages integrate inflammatory signals, respond dynamically to tissue stress, and regulate stem-cell behavior and tissue remodeling pathways, they are well-positioned to influence developmental outcomes in response to environmental perturbations. Their ability to transition between distinct activation states further suggests that macrophages may serve as adaptable cellular intermediaries capable of translating environmental stress signals into long-term changes in tissue organization and developmental patterning.

Within the context of the macrophage-mediated morphological (M3) model proposed in this review, macrophage polarization states may play a particularly important role in determining whether stressed tissues undergo regenerative remodeling or pathological degeneration. In this framework, environmentally induced changes in macrophage activation states and associated small-RNA pathways could influence tissue remodeling responses and potentially contribute to stable epigenetic signaling programs transmitted across generations.

### 3.4. Macrophage Extracellular Vesicles Containing Small RNAs

**Figure 9:**
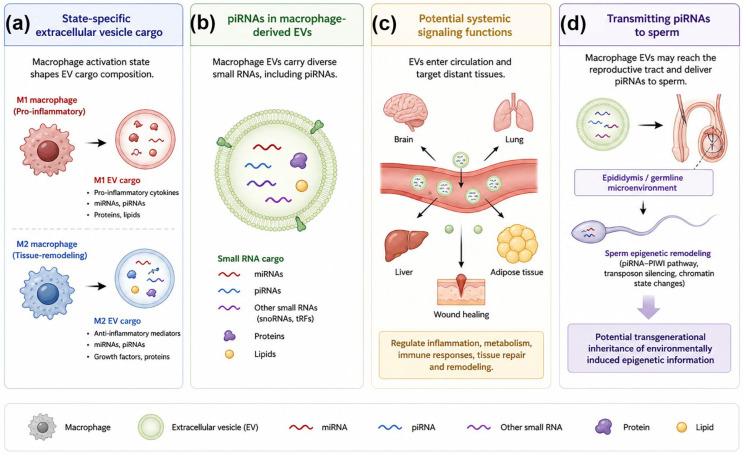
Macrophage extracellular vesicles containing small RNAs. (**a**) Macrophage activation states influence the molecular cargo of extracellular vesicles (EVs), including cytokines, proteins, lipids, and small RNAs. (**b**) Both M1 and M2 macrophages release EVs containing distinct populations of miRNAs, piRNAs, and other non-coding RNAs. (**c**) These EVs can circulate systemically and influence distant tissues involved in inflammation, metabolism, tissue repair, and remodeling. (**d**) The model proposes that macrophage-derived EVs may also reach reproductive tissues and deliver piRNAs to sperm or the epididymal microenvironment, potentially altering PIWI-pathway activity, chromatin states, and transposon repression in germ cells. Such mechanisms could contribute to transgenerational transmission of environmentally induced epigenetic information.

Extracellular vesicles (EVs) have emerged as important mediators of intercellular communication and are increasingly recognized as potential vehicles for systemic transfer of regulatory RNAs, proteins, lipids, and signaling molecules (Figure 9) [192,193,194]. Macrophages are particularly active producers of extracellular vesicles, releasing exosomes and microvesicles in response to inflammatory stimuli, tissue injury, metabolic stress, and developmental signaling environments [195]. These vesicles can travel locally or systemically and influence the behavior of recipient cells by transferring bioactive molecular cargo. Because macrophages integrate environmental stress signals and participate in tissue remodeling processes throughout the body, macrophage-derived extracellular vesicles represent plausible candidates for mediating long-range communication between stressed tissues and distant target organs, including the potential to reach the germline.

One of the most striking features of macrophage-derived extracellular vesicles is that their molecular cargo differs substantially depending on macrophage activation state [196,197,198]. M1 and M2 macrophages release extracellular vesicles containing distinct populations of cytokines, lipids, proteins, metabolites, and small non-coding RNAs [199,200,201]. Vesicles derived from M1 macrophages are enriched for pro-inflammatory signaling molecules that promote inflammatory responses, antimicrobial activity, and antitumor signaling pathways. In contrast, extracellular vesicles released by M2 macrophages are enriched for anti-inflammatory mediators, tissue-remodeling factors, angiogenic signals, and regenerative signaling molecules that support wound healing and tissue repair [202]. These state-specific extracellular vesicle profiles suggest that macrophage activation states shape not only local tissue responses but also systemic communication networks.

Recent small-RNA sequencing studies demonstrate that macrophage-derived extracellular vesicles contain diverse populations of regulatory RNAs, including microRNAs (miRNAs), small nucleolar RNAs (snoRNAs), tRNA-derived fragments (tRFs), and piRNAs [203,204,205,206,207,208]. Importantly, the composition of these small-RNA populations differs between M1 and M2 macrophage-derived vesicles, suggesting selective packaging of regulatory RNAs depending on macrophage functional state [47,204,206,207]. Although the biological functions of macrophage-associated piRNAs remain incompletely understood, these observations raise the possibility that extracellular vesicle-associated piRNAs participate in communication pathways that extend beyond local tissue environments.

The presence of piRNAs within extracellular vesicles is particularly intriguing in the context of epigenetic regulation and transgenerational inheritance. piRNAs possess several properties that make them plausible mediators of stable biological signaling, including resistance to degradation, as discussed above, due to their characteristic 3′ terminal 2′-O-methyl modification and their ability to participate in self-amplifying regulatory pathways through PIWI-associated amplification systems [24]. Packaging within extracellular vesicles may further protect piRNAs from degradation during circulation and facilitate targeted delivery to recipient cells. These properties distinguish piRNAs from many other small RNAs and suggest that extracellular vesicle-associated piRNAs could potentially mediate durable regulatory effects in distant tissues.

Macrophage-derived extracellular vesicles have already been implicated in diverse systemic signaling functions. Studies demonstrate that macrophage EVs influence endothelial cell behavior, stem-cell differentiation, tumor microenvironments, adipose tissue remodeling, fibrosis, and inflammatory signaling networks [52,209,210]. During tissue injury and repair, macrophage-derived vesicles coordinate regenerative responses by transferring cytokines, growth factors, and regulatory RNAs that alter gene-expression programs in recipient cells. Such findings support the broader concept that macrophages function as mobile signaling hubs capable of integrating local environmental information and distributing regulatory signals systemically.

These observations are highly relevant to the possibility that macrophage-derived extracellular vesicles may communicate with germline tissues. Increasing evidence suggests that extracellular vesicles can cross tissue barriers and interact with reproductive tissues, including testes and epididymal environments [211,212,213,214]. In males, epididymal epithelial cells release extracellular vesicles known as epididymosomes that transfer small RNAs and proteins directly to maturing sperm cells during post-testicular maturation [215,216,217]. Environmental stressors such as dietary changes and toxicant exposures alter the small-RNA composition of these vesicles, supporting the idea that extracellular vesicle-mediated communication contributes to environmentally responsive sperm epigenetic remodeling.

**Figure 11:**
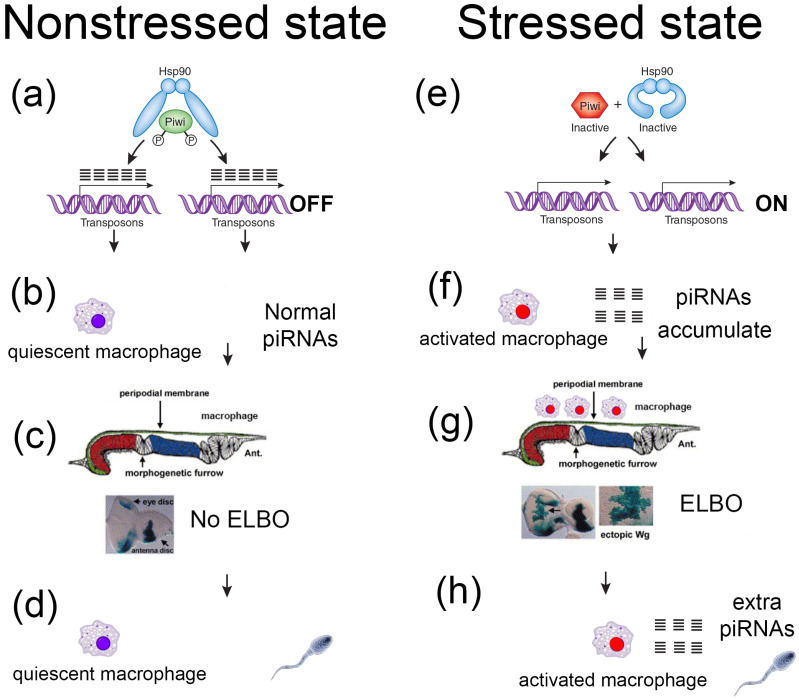
Mechanistic framework for the Macrophage-Mediated Morphological (M3) evolution model. (**a**–**d**) Non-stressed conditions. (**a**) Hsp90 stabilizes Piwi proteins, enabling piRNA-mediated transcriptional silencing of transposable elements. (**b**) Macrophages remain predominantly in a quiescent M2-like state associated with tissue maintenance. (**c**) Limited macrophage migration occurs within the eye imaginal disc. (**d**) No significant transfer of additional piRNAs to germ cells occurs. (**e**–**h**) Stress condition. (**e**) Environmental stress or genetic perturbation of Hsp90 disrupts Piwi activity and partially activates transposable elements. (**f**) Increased transposon transcripts generate additional piRNAs through the cytoplasmic ping-pong amplification pathway. (**g**) Activated macrophages migrate to the eye imaginal disc, engulf apoptotic cells and stimulate local progenitor cells to form ELBO structures through Wg signaling and cytokine release. (**h**) Activated macrophages release small-RNA-containing vesicles that influence germ cells, potentially propagating piRNA populations to sperm and enabling transgenerational transmission of the phenotype. Vertical and diagonal arrows indicate sequential actions. Horizontal arrows indicate direction of mRNA transcription.

Within the context of the macrophage-mediated morphological (M3) model proposed in this review, macrophage-derived extracellular vesicles provide a plausible mechanism for transmission of stress-associated piRNAs from somatic tissues to germline cells (Figure 11). In this framework, macrophages responding to stressed or damaged tissues could acquire altered small-RNA populations during inflammatory and remodeling responses. These macrophages could subsequently release extracellular vesicles enriched for specific piRNAs or other regulatory small RNAs that enter systemic circulation and eventually interact with reproductive tissues or germ cells. Delivery of macrophage-associated piRNAs to sperm or spermatogonial stem cells could potentially alter germline chromatin states, PIWI pathway activity, or transposon repression systems.

This possibility is particularly significant because one of the major unresolved challenges in transgenerational epigenetic inheritance is identifying plausible mechanisms by which environmentally induced somatic signals reach the germline [197]. Macrophage-derived extracellular vesicles offer several conceptual advantages in this regard. Macrophages are highly migratory, environmentally responsive, and capable of integrating signals from damaged tissues throughout the body. Their extracellular vesicles can carry stable regulatory molecules and participate in long-range communication pathways. Furthermore, piRNA-associated pathways possess intrinsic amplification properties that could potentially stabilize environmentally induced signals after delivery to germ cells.

Although direct experimental evidence demonstrating macrophage-to-germline piRNA transfer remains limited, several observations indirectly support this possibility. Environmental stress alters both macrophage activation states and sperm small-RNA populations [218,219,220,221]. Macrophage extracellular vesicles contain regulatory small RNAs capable of altering gene expression in recipient cells [50,222]. Sperm epigenetic states and small-RNA populations are sensitive to environmental exposures [223,224,225,226,227]. Together, these findings suggest that macrophage-derived extracellular vesicles may represent previously underappreciated intermediaries linking environmental stress responses to germline epigenetic remodeling.

Future experimental studies will be necessary to determine whether macrophage-derived extracellular vesicles directly contribute to transgenerational epigenetic inheritance. Important questions include whether macrophage-associated piRNAs can be transferred into germ cells under physiological conditions, whether these RNAs alter sperm epigenetic states or PIWI pathway activity, and whether environmentally induced macrophage-derived vesicle signals persist across generations. Resolving these questions may provide critical insights into how environmentally responsive immune-cell signaling pathways contribute to heritable phenotypic variation.

### 3.5. The ELBO Phenotype as a Model for Stress-Induced Morphological Inheritance

#### The ELBO Phenotype in Drosophila as a Model of Stress-Induced Morphological Plasticity

**Figure 10:**
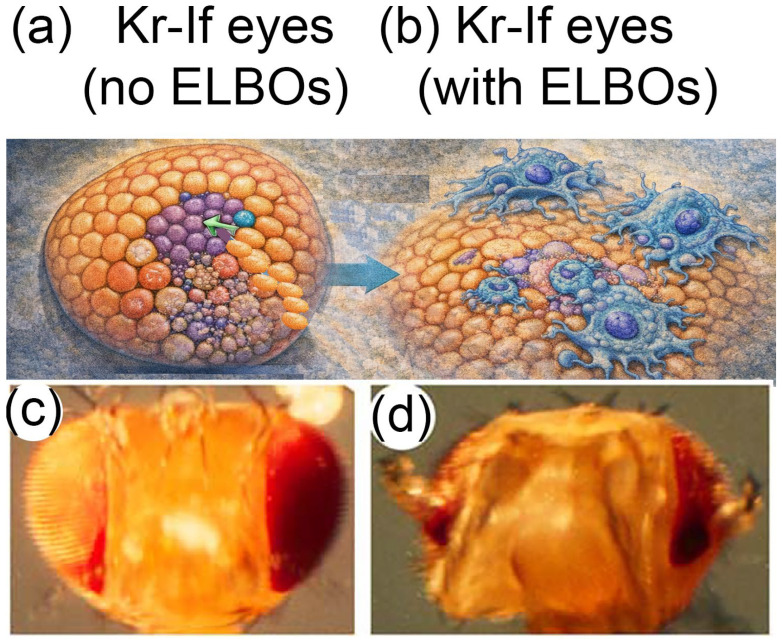
The ectopic large bristle outgrowth (ELBO) phenotype in *Drosophila* and the proposed role of macrophage-like hemocytes in stress-induced morphological remodeling. (**a**) Schematic representation of a *Krüppel* Incomplete facets-1 (*Kr^If-1^*) eye imaginal disc that does not develop ELBOs. Ectopic expression of *Krüppel* (*Kr*) within the eye imaginal disc causes localized degeneration and apoptosis of developing ommatidial precursor cells (“death by indecision”), but limited recruitment of macrophage-like hemocytes is observed. (**b**) Schematic representation of a *Kr^If-1^* eye imaginal disc that develops ELBOs following environmental stress or disruption of Hsp90/piRNA pathway activity. Macrophage-like hemocytes accumulate on the surface of the eye imaginal disc surrounding degenerating tissue. In the proposed macrophage-mediated morphological (M3) model, these hemocytes phagocytose dying cells and release signaling molecules, morphogens, cytokines, or small-RNA-containing extracellular vesicles that alter developmental signaling environments and promote ectopic outgrowth formation. (**c**) Adult *Drosophila* head from a *Kr^If-1^* fly lacking ELBOs, showing relatively intact but reduced eye morphology associated with ectopic *Kr* expression in the developing eye imaginal disc. (**d**) Adult *Kr^If-1^* fly displaying the ELBO phenotype, characterized by ectopic appendage-like bristle outgrowths emerging from the eye region following stress-induced developmental remodeling. Together, these observations suggest that environmental stress, Hsp90/piRNA pathway disruption, and macrophage-mediated tissue remodeling may cooperate to generate stress-induced morphological plasticity in Drosophila.

One of the earliest experimental systems suggesting a potential connection between environmental stress, epigenetic regulation, and heritable morphological variation emerged from studies of the ectopic large bristle outgrowth (ELBO) phenotype in *Drosophila melanogaster* (Figure 10) [53,55,56,112]. This system arose from genetic screens performed in flies carrying the *Krüppel Incomplete facets-1* (*Kr^If-1^*) mutation, in which ectopic expression of the developmental transcription factor Krüppel (Kr) occurs within the developing eye imaginal disc during the third larval instar stage. Because Kr is normally involved in early embryonic segmentation rather than eye differentiation, ectopic expression within eye precursor cells disrupts normal ommatidial development and induces extensive cell death within the eye imaginal disc.

The resulting degenerative process creates a highly abnormal developmental microenvironment within the larval eye disc. Cells expressing ectopic *Kr* fail to differentiate properly and undergo apoptosis in what was previously described as “death by indecision,” reflecting conflicting developmental identity programs within the affected tissue [53,55,56,112]. In most *Kr^If-1^* flies, this developmental disruption produces reduced eye size and abnormal eye morphology without major ectopic structures (Figure 10a,c). However, under conditions of environmental stress or perturbation of Hsp90 function, some flies develop striking ectopic appendage-like outgrowths emerging from the eye surface, termed ectopic large bristle outgrowths (ELBOs) (Figure 10b,d and Figure 11).

ELBO structures resemble proximal appendage-like tissues and are associated with ectopic expression of developmental regulators such as homothorax [53,55,56,112], a proximal appendage specification gene normally not expressed within the eye field [228]. These observations suggested that developmental stress within the eye imaginal disc can trigger inappropriate activation of alternative morphogenetic programs. Importantly, the ELBO phenotype could be induced experimentally through environmental or pharmacological stressors, including inhibition of Hsp90 using geldanamycin [56,121] and exposure to environmental toxicants such as hexavalent chromium [229]. These findings linked stress-induced developmental instability to the emergence of novel morphological phenotypes.

As illustrated in Figure 10a and Figure 11, the eye imaginal discs of *Kr^If-1^* flies lacking ELBOs exhibit localized regions of degenerating cells but relatively limited recruitment of macrophage-like hemocytes to the damaged tissue. In contrast, eye discs from flies that develop ELBOs exhibit extensive accumulation of macrophage-like cells on the surface of the imaginal disc surrounding degenerating regions (Figure 10b and Figure 11). Reanalysis of earlier wg-lacZ reporter gene experiments suggested that many of the cells expressing wingless (wg-lacZ) were likely not intrinsic eye disc cells, but instead migratory hemocyte-like cells associated with damaged regions of the tissue [55]. This observation became central to development of the macrophage-mediated morphological (M3) model proposed in Section 3.6.

The association between macrophage-like hemocytes and ELBO formation raises the possibility that inflammatory or tissue-remodeling responses contribute directly to abnormal morphogenesis. In *Drosophila*, as discussed above, hemocytes are highly migratory phagocytic cells that respond rapidly to damaged tissues and participate in apoptotic cell clearance, extracellular matrix remodeling, and regenerative signaling [166,167,171,230]. Within the ELBO system, hemocytes may initially be recruited to remove dying Kr-expressing cells. However, these macrophage-like cells may also alter local developmental signaling environments by secreting morphogens, cytokine-like factors, extracellular matrix regulators, or small-RNA-containing extracellular vesicles that influence nearby progenitor or stem-like cells within the imaginal disc.

This interpretation is consistent with broader developmental remodeling functions of macrophages described in vertebrate systems. In many tissues, macrophages do not simply remove dead cells but actively coordinate regeneration and tissue restructuring following injury [5]. Similar processes may occur within stressed *Drosophila* eye discs, where macrophage-associated signaling pathways could redirect surviving progenitor cells toward ectopic developmental fates. Such mechanisms could explain how tissue damage and inflammatory responses become coupled to generation of novel morphological structures under stress conditions.

The ELBO system also possesses several features suggesting involvement of epigenetic rather than purely genetic mechanisms. Although ELBO phenotypes could be enriched through selective breeding following stress exposure, penetrance rarely exceeded approximately 80%, the phenotype often displayed striking asymmetry between left and right eyes, and the phenotype disappeared rapidly when selective pressure was removed. These characteristics differ from stable Mendelian mutations and instead resemble environmentally responsive developmental plasticity. Such observations raised the possibility that stress-induced phenotypic variation in the ELBO system involved unstable epigenetic states or environmentally responsive regulatory pathways.

Subsequent studies strengthened the connection between the ELBO phenotype and piRNA pathway regulation. Mutations affecting Piwi-family proteins enhanced ELBO formation, and Hsp90 was shown to function as a critical chaperone for PIWI proteins involved in piRNA biogenesis and transposon repression [53,157,229]. These findings suggested that stress-induced disruption of Hsp90-dependent piRNA pathways might contribute to epigenetic instability and abnormal developmental signaling within stressed tissues.

Within the framework of the M3 model proposed here, the ELBO phenotype may represent more than a localized developmental abnormality. Instead, it may provide a model system illustrating how environmental stress, macrophage activation, tissue remodeling, and small-RNA signaling pathways converge to generate novel morphological outcomes. In this model, macrophages responding to stressed tissues acquire altered regulatory states, potentially involving piRNA-associated pathways, and subsequently influence developmental remodeling processes within damaged tissues. If macrophage-associated small-RNA signals can also enter systemic circulation or germline communication pathways, such mechanisms could theoretically contribute to environmentally responsive transgenerational epigenetic inheritance.

Thus, the ELBO system provides a conceptual bridge linking Hsp90 buffering, piRNA pathway disruption, macrophage-mediated tissue remodeling, and stress-induced morphological plasticity. These observations form a central experimental foundation for the broader hypothesis advanced in this review: that macrophage-associated small-RNA signaling systems may act as intermediaries connecting environmental stress responses to developmental and potentially heritable phenotypic variation.

### 3.6. The Macrophage-Mediated Morphological (M3) Evolution Model

#### 3.6.1. Proposed Mechanism of Macrophage-Mediated Inheritance

The macrophage-mediated morphological (M3) model proposed in this review represents a conceptual framework linking environmental stress, small-RNA regulatory systems, immune-cell plasticity, tissue remodeling, and transgenerational epigenetic inheritance (Figure 11). The central hypothesis of the M3 model is that macrophages or macrophage-like phagocytic cells act as mobile intermediaries that acquire stress-associated molecular information from damaged tissues and subsequently communicate this information systemically, including the potential to reach germline cells through extracellular vesicle-mediated transfer of piRNAs and related small RNAs. In this framework, macrophages function not simply as inflammatory scavenger cells but as environmentally responsive regulators capable of integrating tissue stress signals with epigenetic communication pathways. Importantly, the proposed model remains hypothetical, particularly regarding systemic macrophage-to-germline communication in vertebrates and is intended as a conceptual synthesis integrating findings from developmental biology, immunology, extracellular vesicle biology, and transgenerational epigenetics.

The model begins with environmental or physiological stressors that disrupt normal genome-defense and epigenetic regulatory systems within somatic tissues. Such stressors may include heat shock, oxidative stress, inflammation, environmental toxicants, endocrine-disrupting chemicals, nutritional stress, or pharmacological inhibition of Hsp90. Under normal conditions, Hsp90-dependent pathways stabilize PIWI proteins and maintain efficient piRNA-mediated repression of transposable elements and repetitive genomic sequences. However, environmental stress may overwhelm Hsp90 buffering capacity or directly impair PIWI-associated regulatory systems, resulting in destabilization of transposon repression pathways.

One major consequence of this disruption is activation of transposable elements and increased expression of transposon-derived RNAs [77,78,79,80]. Under normal physiological conditions, transposons are tightly repressed through coordinated actions of piRNA pathways, heterochromatin formation, histone modifications, and DNA methylation systems [12,13,14,15,16,17]. When these repression systems become compromised, transposon transcripts accumulate within stressed cells. This process not only increases genomic instability but also generates abundant RNA substrates that can enter small-RNA amplification pathways, including the ping-pong amplification cycles associated with cytoplasmic PIWI proteins.

Within the M3 model, stress-induced transposon activation is proposed to alter local small-RNA environments in damaged tissues. Increased production of transposon-derived RNAs may stimulate generation of secondary piRNAs and related small RNAs through PIWI-associated amplification systems. Because piRNA pathways possess intrinsic self-amplifying properties, environmentally induced perturbations may produce sustained changes in small-RNA populations even after the initial stress exposure has subsided. These altered small-RNA environments may contribute to epigenetic instability and altered developmental signaling programs within stressed tissues.

The second major component of the M3 model involves macrophage acquisition of stress-associated molecular signals from damaged tissues. Macrophages and macrophage-like hemocytes are highly responsive to tissue injury and rapidly migrate toward sites of apoptosis, inflammation, and cellular stress [231,232,233,234,235,236]. Within damaged tissues, macrophages engulf apoptotic cells, cellular debris, extracellular nucleic acids, and damaged organelles through phagocytic pathways. In doing so, macrophages may acquire stress-associated RNAs, including transposon-derived RNAs and altered populations of piRNAs generated within stressed cells.

Importantly, as discussed above, macrophages themselves possess distinct piRNA expression profiles and environmentally responsive small-RNA regulatory systems [40,47,48,191]. Environmental stress and inflammatory signaling may therefore alter both endogenous macrophage piRNA pathways and the populations of small RNAs acquired from surrounding tissues. Within the M3 framework, macrophages are proposed to function as integrative processing hubs that combine inflammatory signaling pathways, tissue-remodeling programs, and small-RNA regulatory systems into coordinated cellular responses.

Following activation within damaged tissues, macrophages may undergo functional polarization transitions associated with inflammatory or tissue-remodeling phenotypes. These activation states are themselves associated with distinct piRNA expression signatures and extracellular vesicle cargo profiles [40,47,48,191]. Activated macrophages subsequently release extracellular vesicles containing proteins, cytokines, lipids, and small RNAs, including piRNAs and other regulatory non-coding RNAs.

The third major component of the M3 model proposes that macrophage-derived extracellular vesicles mediate systemic transfer of stress-associated small RNAs to distant tissues, including the potential to reach the germline. Extracellular vesicles provide an especially attractive mechanism for such signaling because they protect small RNAs from degradation during circulation and permit targeted delivery into recipient cells [237,238,239]. The characteristic 3′ terminal 2′-O-methyl modification of piRNAs may further enhance their stability during extracellular transport [24,73]. However, direct evidence for physiologically significant macrophage-to-germline transfer of piRNAs in vertebrates remains limited. In vertebrate systems, several important anatomical and physiological barriers—including extracellular vesicle biodistribution constraints, compartmentalization of germline stem-cell niches, and the blood–testis barrier—may substantially restrict direct access of circulating macrophage-derived vesicles to germ cells. Thus, the proposed macrophage-mediated transfer pathway should presently be viewed as a speculative but testable hypothesis.

Within reproductive tissues, macrophage-derived extracellular vesicles may interact with germline-supporting cells, spermatogonial stem cells, developing sperm cells or epididymal maturation environments [240,241,242]. In male reproductive systems, extracellular vesicle-mediated communication already plays important roles in sperm maturation and transfer of regulatory RNAs into sperm during epididymal transit. The M3 model extends this concept by proposing that environmentally responsive macrophage-derived vesicles may contribute additional small-RNA signals capable of altering germline epigenetic states. Importantly, these effects may occur indirectly through intermediary somatic cells within reproductive tissues rather than direct delivery to germ cells themselves.

Once delivered into germ cells, stress-associated piRNAs may influence PIWI pathway activity, transposon repression systems, chromatin organization, or epigenetic regulatory networks. Because piRNA pathways already function naturally in transgenerational transposon silencing and epigenetic inheritance systems in organisms such as *C. elegans* and *Drosophila*, environmentally induced perturbations of these pathways may theoretically generate heritable alterations in gene regulation [27,53,83]. In this context, macrophage-associated piRNAs may function not as entirely novel inheritance mechanisms but rather as modulators of ancient small-RNA-guided genome-defense systems that have been evolutionarily repurposed for environmentally responsive signaling.

A particularly important feature of the M3 model is that it provides a plausible mechanistic bridge between somatic environmental responses and germline epigenetic regulation. One of the major unresolved questions in transgenerational epigenetic inheritance research has been how environmentally induced signals originating in somatic tissues reach germline cells despite developmental barriers separating soma and germline lineages [243,244,245]. Because macrophages are highly migratory, environmentally responsive, and capable of systemic communication through extracellular vesicles, they represent plausible candidates for mediating this transfer of information, although direct experimental validation is still lacking.

The M3 model also offers a potential explanation for why environmentally induced phenotypes often exhibit incomplete penetrance, reversibility, and context-dependent expression. Unlike stable Mendelian mutations, macrophage-mediated signaling pathways may generate probabilistic or dynamic epigenetic states that depend on inflammatory conditions, tissue remodeling responses, and environmental exposures. Such properties are consistent with observations from the ELBO/EBO system in *Drosophila*, where stress-induced morphological phenotypes exhibit partial penetrance, asymmetry, and rapid loss following removal of selective pressure.

Importantly, the M3 model remains hypothetical and requires extensive experimental validation. Critical unanswered questions include whether macrophages directly acquire stress-associated piRNAs from damaged tissues, whether macrophage-derived extracellular vesicles can deliver piRNAs to germ cells under physiological conditions, and whether such transferred RNAs can alter germline epigenetic states across generations. Nevertheless, the convergence of evidence from piRNA biology, macrophage plasticity, extracellular vesicle signaling, developmental remodeling systems, and environmentally induced epigenetic inheritance suggests that macrophage-associated small-RNA pathways represent plausible candidates for mediating environmentally responsive transgenerational signaling mechanisms.

Together, the M3 model proposes an integrated biological framework in which environmental stress destabilizes genome-defense pathways, activates macrophage-mediated tissue remodeling responses, alters small-RNA signaling systems, and potentially permits transmission of stress-associated epigenetic information to future generations.

#### 3.6.2. Experimental Predictions of the M3 Model

The macrophage-mediated morphological (M3) model proposed in this review generates a series of experimentally testable predictions linking environmental stress, macrophage activation, small-RNA signaling, and transgenerational epigenetic inheritance. Because the model integrates concepts from developmental biology, immunology, epigenetics, and small-RNA biology, validation will require multidisciplinary experimental approaches spanning both invertebrate and vertebrate systems. Importantly, the M3 framework predicts not merely correlations between environmental stress and altered germline epigenetic states, but active mechanistic roles for macrophages or macrophage-like phagocytic cells in transmitting environmentally responsive small-RNA signals between somatic tissues and germline-supporting tissues. However, direct macrophage-to-germline transfer of piRNAs remains unproven and should currently be viewed as a speculative but experimentally testable hypothesis.

One of the most direct tests of the M3 model would involve adoptive transfer experiments using macrophage-like cells from environmentally stressed organisms. In the *Drosophila* ELBO/EBO system, hemocytes could be isolated from *Kr^If-1^* larvae that develop ELBO phenotypes following environmental stress or Hsp90 inhibition and transferred into genetically matched larvae that normally do not develop ELBOs. If macrophages contribute causally to the remodeling processes underlying ectopic appendage formation, recipient larvae receiving hemocytes from ELBO-positive donors should exhibit increased frequencies of ELBO development compared with controls receiving hemocytes from non-ELBO donors.

Such experiments could be refined further by isolating specific hemocyte subpopulations associated with damaged eye imaginal discs. For example, fluorescence-activated cell sorting (FACS) approaches can be used to isolate wg-lacZ-positive hemocyte populations enriched near stressed imaginal tissues. These purified macrophage-like cells can then be transferred into recipient larvae to determine whether particular macrophage activation states or signaling populations are sufficient to alter developmental outcomes. If adoptively transferred macrophages induce increased ELBO formation in recipient animals, such findings would provide strong evidence that macrophage-associated remodeling or signaling pathways contribute directly to the phenotype.

An even more provocative prediction of the M3 model is that macrophage-associated states might influence subsequent generations. If macrophages transfer stress-associated piRNAs or other epigenetic signals into germline tissues, offspring derived from recipient animals receiving activated macrophages might themselves exhibit altered phenotypic frequencies or epigenetic states. Such experiments would provide particularly important evidence for macrophage-mediated contributions to transgenerational inheritance pathways, although intermediary somatic tissues may mediate these effects in vertebrates rather than direct delivery to germ cells themselves.

Complementary approaches involve macrophage depletion experiments designed to determine whether macrophages are necessary for stress-induced phenotypic remodeling or epigenetic inheritance. In *Drosophila*, genetic or pharmacological strategies that reduce hemocyte populations could be combined with environmental stress paradigms known to induce ELBO phenotypes. If depletion of hemocytes suppresses ELBO formation despite continued environmental stress exposure or Hsp90 inhibition, this would strongly support active roles for macrophage-like cells in the remodeling process.

Similar approaches could be extended into vertebrate systems. Mouse models allowing conditional depletion of macrophage populations during periods of environmental stress exposure could be used to determine whether macrophages contribute to environmentally induced sperm epigenetic remodeling or transmission of stress-associated phenotypes to offspring. Such studies might involve exposure to toxicants, dietary stress, inflammatory stimuli, or metabolic stressors previously shown to alter sperm small-RNA populations. If macrophage depletion reduces or abolishes environmentally induced germline epigenetic changes, such findings would provide important mechanistic support for the M3 hypothesis. However, vertebrate studies must also account for physiological barriers such as extracellular vesicle biodistribution constraints and the blood–testis barrier, which may limit direct macrophage-derived vesicle access to germ cells.

Another critical experimental direction involves comprehensive small-RNA profiling of macrophages, extracellular vesicles, and germ cells following environmental stress exposure. The M3 model predicts that environmental stress can alter piRNA populations not only within damaged tissues and sperm but also within macrophages responding to stressed environments. Comparative sequencing analyses could therefore examine whether macrophages acquire distinct stress-associated piRNA signatures following environmental perturbation.

Particularly informative experiments would compare small-RNA populations across stressed somatic tissues, macrophages, macrophage-derived extracellular vesicles, epididymal environments, and sperm cells. If shared piRNA signatures emerge across these compartments following environmental stress exposure, this would support the idea that macrophage-associated small RNAs participate in systemic signaling pathways linking stressed tissues to the germline. Time-course experiments could further determine whether macrophage-associated small-RNA changes precede or correlate with alterations observed later in germ cells.

The M3 model also predicts that extracellular vesicles released by activated macrophages should contain environmentally responsive piRNA populations capable of influencing recipient cells. Isolation and characterization of macrophage-derived extracellular vesicles following stress exposure could therefore provide critical insights into potential communication pathways. Experiments tracking fluorescently labeled macrophage-derived extracellular vesicles in vivo might determine whether these vesicles can access reproductive tissues or interact directly with germline-supporting cells. Importantly, these studies would help determine whether macrophage-associated signaling pathways operate through direct germline delivery or through indirect modulation of reproductive microenvironments.

Additional mechanistic studies could examine whether macrophage-associated piRNAs alter chromatin states or PIWI pathway activity in recipient germ cells. For example, germ cells exposed experimentally to macrophage-derived extracellular vesicles could be analyzed for changes in transposon expression, histone modifications, DNA methylation patterns, or PIWI-associated small-RNA populations. Such studies would help determine whether transferred macrophage-associated RNAs produce functional epigenetic consequences within reproductive tissues.

Single-cell transcriptomic and epigenomic approaches may also prove highly informative. These technologies could identify macrophage subpopulations associated specifically with stressed developmental tissues and determine whether distinct inflammatory or small-RNA-associated transcriptional programs emerge during environmentally induced remodeling responses. Similar analyses in reproductive tissues could identify germline-supporting cell populations responsive to macrophage-derived extracellular vesicle signaling.

An especially important prediction of the M3 model concerns transposon activation. Because the model proposes that environmental stress destabilizes Hsp90-dependent piRNA pathways and increases transposon-derived RNA production, experiments should determine whether macrophage activation correlates with increased transposon expression within stressed tissues. If macrophages acquire transposon-derived RNAs or altered piRNA populations from damaged cells, these RNAs might serve as molecular signatures linking somatic stress responses to germline epigenetic changes.

Finally, comparative evolutionary studies may provide additional insight into the plausibility of macrophage-mediated inheritance systems. If analogous interactions between phagocytic immune cells, small-RNA pathways, and transgenerational signaling mechanisms are observed across diverse organisms—including insects, nematodes, and vertebrates—this would support the possibility that macrophage-associated epigenetic communication pathways represent evolutionarily conserved biological mechanisms. At the same time, important evolutionary and anatomical differences between invertebrate hemocyte systems and vertebrate macrophage biology should be considered when extrapolating findings across species.

Together, these experimental predictions provide a roadmap for testing the central claims of the M3 model. Although highly speculative at present, the hypothesis generates clear mechanistic predictions that can be addressed experimentally through macrophage transfer studies, depletion experiments, extracellular vesicle analyses, and small-RNA profiling approaches. Resolving these questions may ultimately determine whether macrophages function not only as immune and tissue-remodeling cells but also as mobile carriers of environmentally responsive epigenetic information capable of influencing future generations.

#### 3.6.3. Limitations and Evolutionary Constraints of the M3 Model

Although the macrophage-mediated morphological (M3) model provides a conceptual framework linking environmental stress, small-RNA signaling, macrophage activation, and transgenerational epigenetic inheritance, several important biological and evolutionary limitations must be acknowledged. In particular, the transition from local hemocyte-mediated remodeling in *Drosophila* imaginal discs to systemic macrophage-mediated signaling pathways in vertebrates represents a substantial mechanistic extrapolation that remains experimentally unproven.

One important distinction involves the evolutionary divergence between invertebrate hemocytes and vertebrate macrophages. *Drosophila* hemocytes are highly migratory macrophage-like cells that interact directly with developing tissues during larval morphogenesis and regenerative remodeling [166,167,168,169,170,171,172]. In contrast, vertebrate macrophages operate within more anatomically compartmentalized tissues and immune microenvironments. Although many phagocytic and inflammatory functions are evolutionarily conserved, the structural organization of vertebrate tissues introduces additional physiological barriers that may restrict direct communication between macrophages and germline cells.

A particularly important constraint is the blood–testis barrier, which creates a highly specialized immunological and molecular microenvironment surrounding developing germ cells in vertebrates [211,212,213,214]. Tight junctions between Sertoli cells restrict movement of circulating molecules and extracellular vesicles into seminiferous tubules, potentially limiting direct access of macrophage-derived extracellular vesicles to developing sperm cells. Similarly, ovarian germline niches are highly compartmentalized and may also restrict penetration of circulating extracellular vesicles into germline microenvironments [7,78,132,133,134,135,136,137,138,139,140,141]. These barriers suggest that any macrophage-associated effects on germline epigenetic states may occur indirectly through intermediary somatic cells rather than through direct macrophage-to-germline delivery.

Additional uncertainties involve extracellular vesicle biodistribution and targeting specificity. Although extracellular vesicles can circulate systemically and influence distant tissues, the efficiency with which macrophage-derived vesicles reach reproductive tissues under physiological conditions remains incompletely understood [211,212,213,214]. Many extracellular vesicles are rapidly cleared from circulation or accumulate preferentially within reticuloendothelial tissues such as the liver and spleen. Thus, whether sufficient quantities of macrophage-derived piRNAs could realistically access germline-supporting tissues to induce stable epigenetic changes remains unknown.

Another limitation concerns the currently limited direct evidence for macrophage-associated piRNA transfer into vertebrate germline systems. Existing studies demonstrate that macrophages possess distinct piRNA expression profiles, that macrophage-derived extracellular vesicles contain small RNAs, and that sperm epigenetic states respond to environmental exposures [211,212,213,214]. However, a direct mechanistic linkage between these observations has not yet been established experimentally. At present, no studies have definitively demonstrated physiologically significant transfer of macrophage-derived piRNAs into vertebrate germ cells followed by stable transgenerational inheritance of altered phenotypes.

The M3 model also does not exclude alternative mechanisms for environmentally responsive epigenetic inheritance. Other small-RNA pathways, including tRNA fragments (tRFs), microRNAs, endogenous siRNAs, chromatin remodeling systems, metabolic signaling pathways, and endocrine signaling mechanisms, may contribute independently or cooperatively to environmentally induced inheritance phenomena. Macrophage-associated signaling may therefore represent only one component of a broader network of environmentally responsive intercellular communication pathways.

Importantly, the proposed model should therefore be viewed as a conceptual and experimentally testable framework rather than an established biological mechanism. The primary value of the M3 hypothesis is that it integrates previously disconnected observations from developmental biology, macrophage biology, extracellular vesicle signaling, piRNA pathways, and transgenerational epigenetics into a unified mechanistic model that generates clear experimental predictions. Future studies will be required to determine whether macrophage-mediated small-RNA signaling contributes directly to germline epigenetic regulation or whether the observed associations instead reflect indirect or parallel stress-response pathways.

## 4. Discussion and Summary

The studies reviewed here support an emerging conceptual framework in which macrophages, small-RNA pathways, and environmental stress responses may intersect to influence developmental plasticity and potentially transgenerational epigenetic inheritance. Although piRNAs were originally characterized as germline genome-defense molecules that repress transposable elements, accumulating evidence now suggests that piRNA-associated pathways extend beyond the germline and participate in broader regulatory networks involving immune-cell function, inflammatory signaling, tissue remodeling, and environmentally responsive epigenetic regulation [246,247,248,249]. At the same time, macrophages are increasingly recognized not only as innate immune cells but also as central regulators of developmental remodeling, regeneration, and systemic signaling [186,250,251,252,253]. The macrophage-mediated morphological (M3) model proposed in this review attempts to integrate these fields into a unified hypothesis linking environmental stress to heritable phenotypic variation (Figure 11).

The *Drosophila* ELBO/EBO system provides an especially provocative experimental framework for exploring these ideas. The combination of stress-induced morphological variation, Hsp90-dependent buffering, piRNA pathway involvement, macrophage recruitment to damaged imaginal discs, and partial multigenerational transmission creates a unique model linking developmental instability to epigenetic regulation [53,54,55,56]. Reinterpretation of wg-lacZ-positive cells as macrophage-like hemocytes associated with stressed tissues further strengthens the possibility that immune-cell-mediated remodeling processes contribute directly to ectopic morphogenesis. Although definitive causal evidence remains lacking, these observations suggest that macrophage-associated signaling pathways may influence developmental outcomes in stressed tissues.

A useful additional vertebrate model that deserves mention is the zebrafish (*Danio rerio*), although a detailed discussion was not included concerning this because of space limitations. Zebrafish are widely used for studying developmental biology, innate immunity, regeneration, and epigenetic regulation because embryos develop externally and remain optically transparent during early embryogenesis, allowing direct visualization of macrophage migration, tissue remodeling, and germline development in vivo [254]. Importantly, zebrafish possess conserved PIWI-family proteins and functional piRNA pathways involved in transposon repression and germline maintenance [255]. Zebrafish macrophages and neutrophils can also be imaged dynamically during tissue injury and regeneration using fluorescent reporter systems, making this organism particularly well suited for testing interactions between inflammatory signaling, extracellular vesicle biology, and small-RNA-mediated developmental remodeling [256]. In addition, zebrafish have emerged as an important model for studying environmentally induced epigenetic effects, including toxicant-associated alterations in small-RNA pathways and transgenerational phenotypes [257,258]. Future studies using zebrafish could therefore provide an experimentally tractable vertebrate system for evaluating key predictions of the M3 model, particularly whether environmentally responsive macrophage-associated small RNAs influence reproductive tissues or developmental plasticity across generations.

The growing evidence for distinct piRNA signatures in M1 and M2 macrophages further supports the plausibility of this framework [40,47,48,191]. If macrophage polarization states are regulated in part through piRNA-associated epigenetic pathways, then environmental stressors that alter macrophage activation states may simultaneously reshape macrophage-associated small-RNA populations. The discovery that macrophage-derived extracellular vesicles contain piRNAs and other regulatory small RNAs introduces additional mechanisms through which immune cells could participate in long-range epigenetic communication networks [40,47,48,191].

The comparison between piRNA pathways and alternative tRNA fragment (tRF) inheritance models also highlights important mechanistic considerations. While tRFs may participate in environmentally responsive sperm signaling pathways, piRNA systems possess several features that may make them particularly well suited for stable transgenerational inheritance, including self-amplifying ping-pong cycles, integration with chromatin-silencing systems, and enhanced biochemical stability through 2′-O-methylation [23,123,259,260,261,262,263,264]. These properties provide plausible mechanisms through which environmentally induced small-RNA signals might persist across developmental stages or generations.

Despite these intriguing connections, the M3 model remains highly speculative, and many critical questions remain unresolved. Direct evidence demonstrating transfer of macrophage-associated piRNAs into germ cells is currently lacking. It is also unclear whether macrophage-derived extracellular vesicles can reach reproductive tissues under physiological conditions at sufficient levels to alter germline epigenetic states. Furthermore, distinguishing true transgenerational inheritance from indirect developmental or parental effects remains experimentally challenging. Future studies involving macrophage depletion, adoptive transfer experiments, extracellular vesicle tracking, and integrated small-RNA profiling across somatic and germline compartments will therefore be essential.

More broadly, the M3 model raises the possibility that immune systems may participate in forms of biological memory extending beyond classical host defense. Macrophages already exhibit forms of trained immunity in which prior environmental exposures alter future inflammatory responses through epigenetic remodeling pathways [265,266,267,268,269]. It is conceivable that some of these environmentally responsive regulatory systems evolved not only to coordinate tissue repair and inflammation but also to integrate information about environmental stress conditions across generations.

If correct, such mechanisms would have important implications for environmental health, developmental biology, reproductive medicine, and evolutionary theory. Environmental toxicants, metabolic stress, inflammation, and aging-related processes may influence not only somatic physiology but also heritable epigenetic states through immune-cell-mediated signaling pathways. Understanding these interactions could provide new insights into how environmental exposures contribute to disease susceptibility, developmental variation, and adaptive physiological responses across generations.

Ultimately, the M3 hypothesis should be viewed as a testable conceptual framework rather than an established mechanism. However, the convergence of evidence from piRNA biology, macrophage plasticity, extracellular vesicle signaling, developmental remodeling systems, and environmentally responsive epigenetic inheritance suggests that further investigation of macrophage-associated small-RNA pathways may reveal previously unrecognized connections between environmental stress, immune signaling, and heredity.

Finally, although the M3 model remains highly speculative, its potential relevance to human health warrants further investigation. Human epidemiological and clinical studies increasingly suggest that environmental exposures—including endocrine-disrupting chemicals, PFAS, air pollution, psychosocial stress, metabolic disease, and inflammation—can alter sperm epigenetic states and small-RNA populations. At the same time, macrophages are now recognized as central regulators of chronic inflammatory diseases, aging-associated tissue remodeling, fibrosis, metabolic dysfunction, and cancer. These observations raise the possibility that environmentally responsive immune-cell signaling pathways could contribute indirectly to reproductive and developmental outcomes in humans. Importantly, the M3 model does not propose Lamarckian inheritance of complex acquired traits but rather suggests that stress-responsive small-RNA and inflammatory signaling systems may influence probabilistic developmental and epigenetic states across generations. If aspects of this framework prove correct, they could have important implications for environmental risk assessment, reproductive medicine, developmental toxicology, and public health, particularly in understanding how chronic environmental stressors influence disease susceptibility and developmental trajectories in descendants.

## 5. Testable Hypotheses, Alternative Mechanisms, and Future Directions

The macrophage-mediated morphological (M3) model proposed in this review should be viewed as a conceptual and experimentally testable framework rather than an established biological mechanism. The model integrates observations from small-RNA biology, macrophage plasticity, extracellular vesicle signaling, developmental remodeling, and transgenerational epigenetics into a unified hypothesis linking environmental stress to heritable phenotypic variation. Importantly, several key aspects of the model remain speculative and require direct experimental validation.

One central prediction of the M3 hypothesis is that environmentally stressed tissues generate altered piRNA populations that can be acquired by macrophages or macrophage-like phagocytic cells during tissue remodeling responses. This prediction can be tested through comparative small-RNA profiling of stressed tissues, macrophages, macrophage-derived extracellular vesicles, and reproductive tissues following defined environmental exposures. Demonstration of shared or sequentially propagated piRNA signatures across these compartments would provide important support for the proposed signaling pathway.

A second major prediction is that macrophages actively contribute to developmental remodeling and potentially to transgenerational signaling. In the *Drosophila* ELBO/EBO system, adoptive transfer of activated hemocytes from ELBO-positive larvae into unstressed recipients could determine whether macrophage-associated states contribute causally to ectopic morphogenesis. Complementary depletion studies in both invertebrate and vertebrate systems could test whether macrophages are required for environmentally induced epigenetic remodeling or inheritance of phenotypes.

Another important experimental prediction is that macrophage-derived extracellular vesicles contain environmentally responsive piRNAs capable of influencing recipient cells. Future studies using fluorescently labeled extracellular vesicles, lineage tracing, single-cell transcriptomics, and reproductive tissue imaging will be essential to determine whether macrophage-derived vesicles can access germline-supporting tissues under physiological conditions. Importantly, vertebrate systems contain substantial anatomical barriers, including the blood–testis barrier and compartmentalized germline niches, which may restrict direct macrophage-to-germline signaling. Thus, macrophage-associated extracellular vesicles may act indirectly through intermediary somatic cells within reproductive tissues rather than through direct delivery into germ cells themselves.

At the same time, several alternative mechanisms could potentially explain environmentally responsive inheritance phenomena independently of the M3 model. Other small-RNA pathways, particularly tRNA-derived fragments (tRFs), microRNAs, and endogenous siRNAs, have also been implicated in environmentally induced sperm signaling and intergenerational phenotypes [16,17,18,19]. Endocrine signaling, metabolic alterations, inflammatory cytokines, microbiome-associated metabolites, and stress-induced chromatin remodeling pathways may similarly influence germline epigenetic states without requiring direct macrophage-mediated RNA transfer. Furthermore, environmentally induced developmental effects observed in offspring may sometimes reflect altered parental physiology, in utero environments, or indirect parental behavioral influences rather than true transgenerational epigenetic inheritance.

The possibility also remains that macrophage-associated piRNA signaling functions primarily in local tissue remodeling rather than in systemic inheritance pathways. In this alternative interpretation, macrophages may influence stress-induced developmental plasticity and regeneration within damaged tissues without directly transmitting epigenetic information across generations. Such a model would still have important implications for developmental biology and inflammatory remodeling while avoiding stronger claims regarding germline inheritance.

Importantly, the M3 model does not propose classical Lamarckian inheritance of complex acquired characteristics. Rather, it suggests that environmentally responsive small-RNA and inflammatory signaling systems may alter probabilistic developmental and epigenetic states that influence susceptibility, plasticity, or phenotypic variability in descendants. These effects would likely operate within the context of existing genetic architectures and developmental constraints rather than functioning as deterministic inheritance systems.

Ultimately, distinguishing among these alternative mechanisms will require rigorous experimental studies integrating developmental biology, immunology, epigenetics, extracellular vesicle biology, and reproductive physiology. Regardless of whether the complete M3 model proves correct, the convergence of evidence reviewed here strongly suggests that immune signaling pathways, small-RNA systems, and environmental stress responses interact more extensively than previously appreciated. Understanding these interactions may provide important new insights into how environmental experiences influence development, disease susceptibility, and biological inheritance across generations.

## Data Availability

No new data were created or analyzed in this study.

## Abbreviations

The following abbreviations are used in this manuscript:

ELBO	Ectopic large bristle outgrowth (epigenetic phenotype in *Drosophila*; aka EBO)
Hsp90	Heat-shock protein 90
RdRP	RND dependent RNA polymerase
PIWI	P-element-induced WImpy testis
piRNA	PIWI RNA
miRNA	Micro RNA
siRNA	Small interfering RNA
piRISC	piRNA PIWI complex
M1	M1 macrophage (generally pro-inflammatory)
M2	M2 macrophage (generally anti-inflammatory/pro-fibrotic)
M3	Macrophage-mediated morphological evolution model
tRF	tRNA fragment
RDDM	RNA-directed DNA methylation
RNAe	RNA-induced epigenetic silencing
H3K9me	Histone 3 lysine 9 methylation (repressive mark)
scnRNA	Scan RNA (ciliates use these for somatic nucleus DNA elimination)
LPS	Lipopolysaccharide
EV	Extracellular vesicle
*Kr*	*Krueppel* (*Drosophila* segmentation gene)
